# Quantifying changes in ambient NO_x_, O_3_ and PM_10_ concentrations in Austria during the COVID-19 related lockdown in spring 2020

**DOI:** 10.1007/s11869-022-01232-w

**Published:** 2022-07-22

**Authors:** C. Staehle, M Mayer, B. Kirchsteiger, V. Klaus, J. Kult-Herdin, C. Schmidt, S. Schreier, J. Karlicky, H. Trimmel, A. Kasper-Giebl, B. Scherllin-Pirscher, H. E. Rieder

**Affiliations:** 1 Institute of Meteorology and Climatology, Department of Water, Atmosphere and Environment, University of Natural Resources and Life Sciences, Vienna, Austria; 2 Institute of Chemical Technologies and Analytics, TU Wien, Vienna, Austria; 3 Department of Atmospheric Physics, Faculty of Mathematics and Physics, Charles University, Prague, Czech Republic; 4 Zentralanstalt für Meteorologie und Geodynamik (ZAMG), Vienna, Austria

**Keywords:** Air quality, COVID-19, Meteorological filtering, Traffic reduction

## Abstract

During spring 2020, unprecedented changes in local and regional emissions have occurred around the globe due to governmental restrictions associated with COVID-19. Many European countries including Austria issued partial curfews or stay-at-home order policies, which have impacted ambient air quality through reductions in non-essential transportation and energy consumption of industrial sites and work places. Here, we analyse the effect of these measures on ambient concentrations of nitrogen oxides (NO_x_), ozone (O_3_) and particulate matter (PM_10_) during the first nationwide lockdown in Austria (16.03.2020 to 14.04.2020). To ensure a robust analysis, the Austrian domain is divided into four individual subsectors contingent on regional climate. For air quality analysis a novel method is applied for filtering days with comparable weather conditions during the 2020 lockdown and spring 2017 to 2019. In general, our analysis shows decreasing pollutant concentrations, although in magnitude dependent on pollutant and regional subdomain. Largest reductions are found for NO_x_ reaching up to −68% at traffic sites reflecting the substantial decrease in non-essential transport. Changes in the O_3_ concentrations at background sites show a rather weak response to NO_x_ declines varying between roughly −18 to +8% for both the median and the upper tail of the distribution. Occasional site level increases in O_3_ concentrations can be attributed to comparably weak titration during night-time. PM_10_ concentrations show the smallest response among air pollutants, attributable to manifold precursor sources not affected by the lockdown measures. However, our analysis indicates also a shift of PM_10_ distributions at traffic sites closer to distributions observed at background sites.

**Supplementary Information:**

The online version contains supplementary material available at 10.1007/s11869-022-01232-w.

## Introduction

Ambient air quality is determined not only by local emissions, but also by long-range transport of pollutants, prevailing meteorological conditions and deposition processes (e.g. He et al. 2017; Jacob and Winner 2009; Kroll et al. 2020; Liu et al. 2020; Ordóñez et al. 2005; Pearce et al. 2011; Vieno et al. 2010). Hence, it is necessary to disentangle different processes to quantify the individual contributions to ambient air pollution burdens. The impact of emission reductions is of specific interest, given that despite much progress in emission abatement in recent years, ambient air quality targets are still frequently exceeded in many countries worldwide (e.g. EEA 2020; Fleming et al. 2018; Shaddick et al. 2020; WHO 2016).

During spring of 2020, unprecedented changes in economic activity and therefore emissions of air pollutants (e.g. Barré et al. 2020; Chen et al. 2020; Dacre et al. 2020; Goldberg et al. 2020; Guevara et al. 2021; Huang and Sun 2020; Keller et al. 2021; Kroll et al. 2020; Le Quéré et al. 2020; Lee et al. 2020) have occurred on a global scale due to measures imposed by governments in order to curb the spread of SARS-CoV-2 and subsequently of the coronavirus disease 2019 (hereinafter referred to as COVID-19). Temporary office, factory and store closures, and (partial) curfews or stay-at-home order policies have induced reductions in non-essential transportation and energy consumption and hence impacted ambient air quality (e.g. Diffenbaugh et al. 2020; Le Quéré et al. 2020; Venter et al. 2020; Wang et al. 2020).

Since the beginning of the COVID-19 pandemic, several lockdowns have been imposed in many countries around the globe. Particularly, during March and April 2020, many regions experienced lockdowns with a duration of several weeks. Here, we focus on the effects of the first nationwide COVID-19 lockdown in Austria (16.03.2020–14.04.2020; hereinafter referred to as initial shutdown period 2020 (ISDP20)) that affected mobility and industrial activities and associated emissions. During ISDP20, the largest traffic reductions, compared to subsequent lockdowns in 2020–2021, were observed in Austria, and also neighbouring countries had issued simultaneous lockdown orders affecting also regional emission patterns and transboundary pollutant transport.

The reduction of the traffic volume should reflect in substantially reduced nitrogen oxide (NO_x_ = NO + NO_2_) concentrations, since road transportation is responsible for about 50% of total Austrian NO_x_ emissions (Anderl et al. 2021). NO_x_ are important precursors for tropospheric ozone (O_3_) and secondary aerosol formation (e.g. Lelieveld and Dentener 2000; Monks et al. 2015). The second relevant precursor group for surface O_3_ is volatile organic compounds (VOCs) originating from both anthropogenic and biogenic sources (AVOC and BVOC, respectively). Besides the availability of precursors, the ratio of NO_x_ to VOC determines ambient O_3_ production which depends also on solar radiation and ambient temperature affecting BVOC emissions as well as kinetic reaction rates (e.g. Barnes and Fiore 2013; Shen et al. 2015; Steiner et al. 2006). In contrast to NO_x_, VOC emissions are not expected to be affected noticeably during ISDP20 in Austria given that traffic contributes less than 5% to total Austrian non-methane volatile organic compound (NMVOC) emissions (Anderl et al. 2021). From an environmental health perspective particulate matter (PM), either directly emitted or formed via chemical processes in the atmosphere is the air pollutant most relevant for human health (WHO 2013, 2016). As for NO_x_, PM emissions are affected by traffic reductions but the resulting changes in PM concentrations are expected to be much smaller and regionally more variable due to differences in the source apportionment of PM (Anderl et al. 2021).

A large body of literature has emerged during the last months documenting the impact of lockdown measures on ambient air quality around the world (for a collection of research articles we refer the interested reader to e.g. https://amigo.aeronomie.be/index.php/covid-19-publications/). Here, we present a study adding to this effort by detailing the effects of stay-at-home orders and other restrictions on ambient air quality in Austria, disentangled from meteorological variability, which has been shown to be of uttermost importance to cleanly attribute changes in ambient air quality to changes in local/regional emissions (e.g. Li et al. 2019; Seo et al. 2018). In this study, we present a novel approach that categorises observations based on meteorological key variables promoting atmospheric formation and dispersion of air pollutants. This allows us to quantitatively estimate the impact of emission changes during ISDP20 and separate it from meteorological conditions. To this end, we analyse observations of NO_x_, O_3_ and PM_10_ available from the Austrian national air quality monitoring network, maintained jointly by the Environment Agency Austria (Umweltbundesamt) and the nine provincial governments. The analysis of local/regional/national changes in Austrian ambient air quality during the ISDP20 can serve also as a test case regarding changes to air pollution levels expected over the coming years, assuming the implementation of more stringent abatement measures underpinning near-term NO_x_ and PM emission scenarios.

## Data & methods

### Study region

**Fig. 1:**
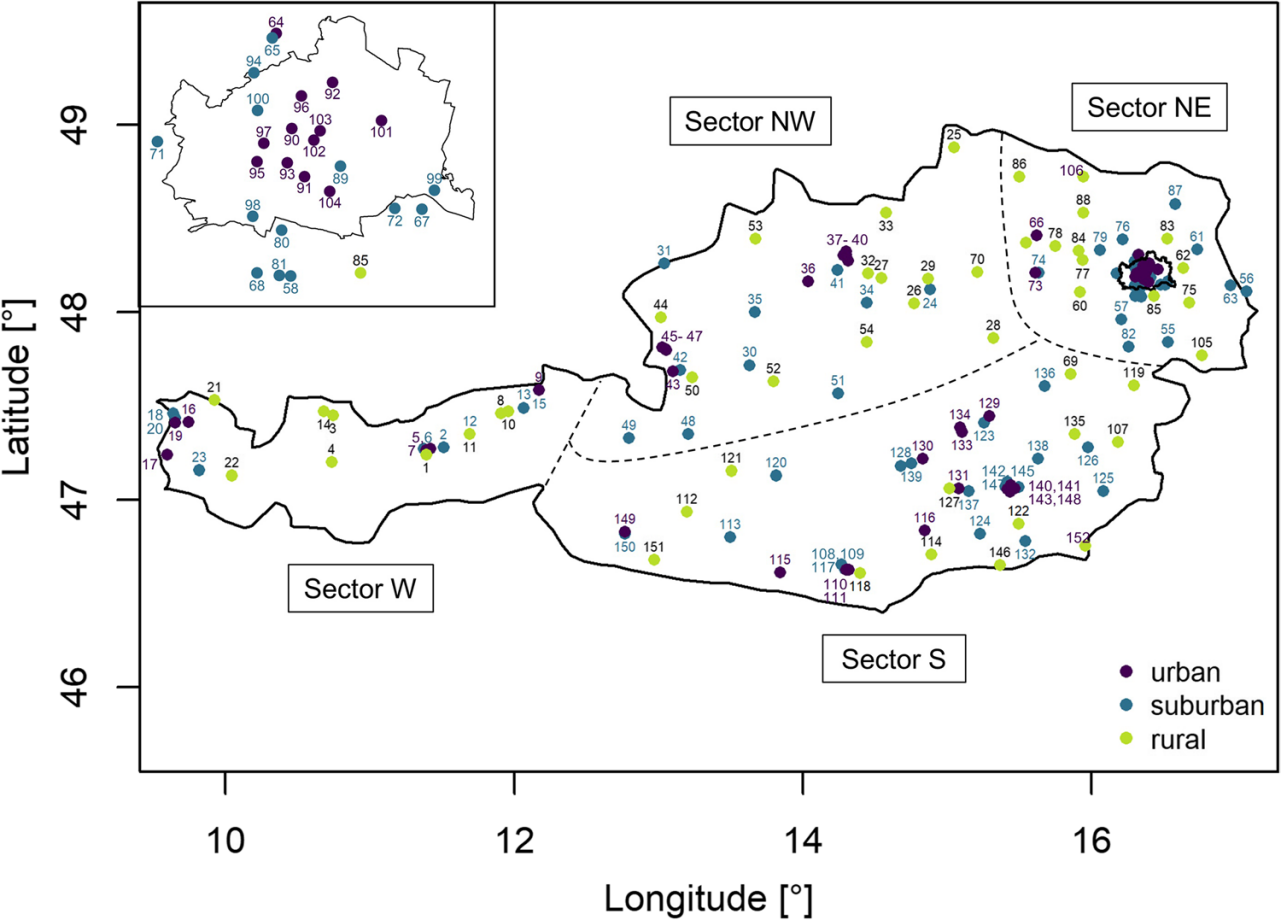
Overview of measurement site locations in the 4 regional subdomains (separated by dashed lines). For convenience, the Vienna metropolitan region (included in the NE sector) is highlighted in the blow-up in the upper left corner. Numbers near station locations are site identifiers (for details (location, sector, classification type and area, as well as pollutants monitored) see supplemental Table S1)

Austria’s topography is rather complex, characterised by the Alps, the Alpine foothills and Eastern lowlands. To account for the differences in terrain and regional meteorological conditions, we define four regional domains referred to as sectors in our analysis. These sectors are broadly separating regions with mountainous, hilly and flat terrain. Sector west (W) comprises Austria’s high-alpine provinces of Vorarlberg and northern Tyrol. The north-western sector (NW) combines the alpine foothills of Upper Austria as well as the hilly western parts of Lower Austria and the parts of Salzburg and Styria north of the central Alpine ridge. The north-eastern sector (NE) comprises Vienna, the central and eastern parts of Lower Austria and the northern part of Burgenland. The southern sector (S) includes Carinthia, eastern Tyrol, parts of Salzburg and Styria south of the central Alpine ridge, a small south-eastern part of Lower Austria and southern Burgenland. Borders between the sectors are drawn along the Alpine main crest, which is a significant meteorological divide between climatic regions more frequently affected by oceanic air masses in the north and Mediterranean air masses in the south. The W and NW sectors are more strongly influenced by air masses from the North Atlantic sector, while sector S shows stronger Mediterranean influence and sector NE shows continental climate characteristics. The location of individual measurement sites of the Austrian air pollution monitoring network in the defined domains (sector W, sector NW, sector NE, sector S) is shown in Fig. 1.

### Air quality data

Half-hourly observational data of NO_2_, NO, O_3_ and PM_10_ available from the national ambient air quality monitoring network are the basis of our analysis. Excluding industrial monitoring sites and sites above 1500 m altitude, we focus our analysis on data from a total of 152 monitoring sites. In order to compare 2020 air pollution levels with those of previous years, and to take into account the overall declining tendencies of air pollution, we use data covering the period from 01.01.2017 to 26.04.2020.

Monitoring sites are classified according to the predominant emissions (background, traffic) and the characteristic of the area (rural, suburban, urban) (Dijkstra and Poelman 2014). Information on all sites, including information on site-specific observed pollutants, and the classification as mentioned above is provided in Table S1 in the supplemental material.

It is well understood that in situ observations of short-lived pollutants such as NO_x_ show a significant small-scale spatial variability depending on the distance from emission sources such as road transport. In urban areas (e.g. Vienna), it is difficult to derive representative areal averages from individual point observations. Therefore, we use upper level (roughly 100 m above ground) measurements of NO_2_ from the DOAS-VINDOBONA network (Schreier et al. 2020) consisting of 3 Multi AXis Differential Optical Absorption Spectroscopy (MAX-DOAS) instruments. These data are concentrations retrieved approximately 150 m above ground over a horizontal distance of approximately 9 km and allow us to quantify regional changes of NO_2_ for the Vienna metropolitan region.

### Meteorological filtering method

To account for the meteorological influence on pollution levels and to discriminate it from the impact of emission changes on the observed pollutant concentrations, we apply a novel meteorological filtering method (MFM). The MFM consists of four steps: (i) we divide the geographic domain into regions (“sectors”) with similar climatological characteristics within (as described in Section 2.1), (ii) we define two weather categories that are associated with high pollutant concentrations (one category for NO_x_ and O_3_ and one for PM_10_), (iii) we sample an equal number of days in each weather category during the study period, defined as 01.03. to 30.04 for each year in 2017 to 2019, and the ISDP20, and (iv) we quantify pollutant concentration changes by comparing concentrations measured in the study period with those of a defined reference period. Additionally, to address the weekday-weekend cycle of air pollution, we distinguish between weekdays and weekends and perform the MFM separately for both cases.

The MFM is based on two meteorological data sets: (i) hourly data obtained from the Integrated Nowcasting through Comprehensive Analysis (INCA) archive (1×1 km grid resolution) (Haiden et al. 2011) of the Austrian meteorological service (ZAMG) and (ii) a large-scale weather classification for the Austrian domain according to the “European Cooperation in Science and Technology” Action 733 (COST 733) weather classification scheme (WLKC733) (Philipp et al. 2010). Meteorological covariates provided in the data sets include global radiation, temperature at 2 m above ground, precipitation amount and wind speed in northward and eastward direction (all from hourly-resolved INCA data, for grid-cells matched with monitoring sites), as well as cyclonicity at 925 hPa and 500 hPa, and a large-scale moisture indicator and flow classification (all from WLKC733 on daily basis).

With the MFM, we aim for selecting meteorological conditions that favour high pollutant concentrations i.e. conditions that are dominated by local emissions rather than background concentrations. Therefore, we denote fair-weather conditions (i.e. conditions promoting formation of O_3_) as “A”-days, applied for NO_x_ and O_3_ analysis, and classify all days not belonging to this category as “C”-days used for the PM_10_ analysis (see below). The classification of “A”- and “C”-days is determined by the daily temperature range, defined as the difference between daily maximum and minimum temperature, and the daily total global radiation sum calculated for each site during the study period. Fair-weather conditions (“A”-days) were selected based on the following criteria: (i) days exceeding the 60th percentile of the diurnal temperature range at site level in the study period; (ii) days exceeding the radiation threshold of 80% of the empirical maximum daily clear sky global radiation calculated by a polynomial fit, following the procedure of Mayer et al. (2022) who plot the annual cycle of the daily sums of the total global radiation for the 30-year period 1990–2019 and fit a polynomial of 5th order to the envelope. This envelope-function approximates the maximum feasible total global radiation for each day of the year. “Clear sky” conditions are assumed if the observed total daily global radiation reaches 80% of the polynomial fit on a specific day. While the first requirement aims to eliminate days with strong advection, as higher wind speed typically reduces the diurnal temperature range, the latter restricts the selection to days with clear sky conditions. If more than 70% of the available sites within a regional domain fulfilled both criteria, the day is considered to be governed by fair-weather conditions and classified as an “A”-day. We note that we compute the temperature range instead of using direct wind information as the resolution of the gridded meteorological data set (1×1 km) bears problems for wind data in alpine terrain; similarly, we use global radiation instead of cloud cover as the gridded product for radiation is based on the Austrian station network of ZAMG and only few sites provide direct information on cloud amount.

From the total number of “A”-days identified for each sector, only weekdays were considered for further analysis due to strong variations in pollutant emissions/concentrations between weekdays and weekends (e.g. Castell-Balaguer et al. 2012; Schipa et al. 2008; Sicard et al. 2020b). To ensure a comparable number of days per year in the total sample, we draw “A”-days with a random sampling approach, individually for each subdomain, with the sample size equal to the smallest number of fair-weather days in a single year and subdomain, respectively. Additionally, we ensure in the sampling that the annual sets of “A”-days are consisting of days from both March and April, to avoid random biases. As a consequence of this requirement, we had to exclude data from 2018 from further analysis because no fair-weather days were identified throughout all of March in that year. The final samples consist of 7 fair-weather weekdays per sector for ISDP20 and the corresponding time periods in 2017 and 2019 (for details about the sample days per sector see supplemental Table S2).

For the analysis of PM_10_ concentrations in a first step “C”-days were selected. Thereafter, the sample is further reduced by selecting only “C”-days indicative of calm winds and no dominant wind flow direction, according to the WLKC733 classification. This approach promotes the selection of “C”-days with stagnant weather conditions and hence elevated PM_10_ concentrations. To ensure consistency with the NO_x_/O_3_ sample 2018 data is not considered. Additionally, days from 27.03.2020 to 29.03.2020 have been excluded from the sample as long-range transport of dust from central Asia has induced high PM_10_ concentrations in the South-East of Austria and other European countries during that period (Šikoparija 2020). In analogy to the NO_x_ and O_3_ sample, the amount of days is determined by the smallest amount of “C”-days in a single year and subdomain, respectively. Hence, the final PM_10_ sample has been filtered to contain 6 weekdays per year and sector (for details about the PM_10_ sample days per sector see supplemental Table S3).

### Quantifying concentrations changes

For the quantification of pollutant concentrations, we (i) focus the analyses on (half-hourly) data selected through the MFM and (ii) contrast data from 2020 and 2019. We do however no longer include data for 2017 since ambient NO_x_ concentrations declined from 2017 to 2019 on average 7% per year, due to the general vehicle fleet renewal (Spangl and Nagl 2020). Furthermore, we assume that changes in NO_x_ and PM_10_ emissions during ISDP20 did not only affect pollutant concentrations but also the overall shape of the probability distributions. Therefore, we analyse several quantiles of interest: for NO_x_ and PM_10_ the 50th (Q50) and 90th (Q90) quantile, and for O_3_ additionally also the 10th quantile (Q10). As photochemistry is of uttermost importance for ozone, we derive quantiles for daytime data only (defined as 06:00 am–06:00 pm CET). For NO_x_ and ozone, we assume a measurement error of ±0.75 μg/m^3^, thus we treat all absolute differences smaller than 1.5 μg/m^3^ between ISDP20 and comparison periods as non-distinguishable (i.e. zero). For the Vienna metropolitan region, we additionally quantify changes in daytime NO_2_ and HCHO concentrations, available from MAX-DOAS measurements for MFM selected days. For individual monitoring sites, we evaluate mean daily cycles of NO, NO_2_ and O_3_ to identify changes in the temporal variation of pollutant concentrations and particularly to differentiate between day- and night-time effects as well as titration in the case of O_3._

## Results

### The evaluation of the meteorological filtering method

**Fig. 2:**
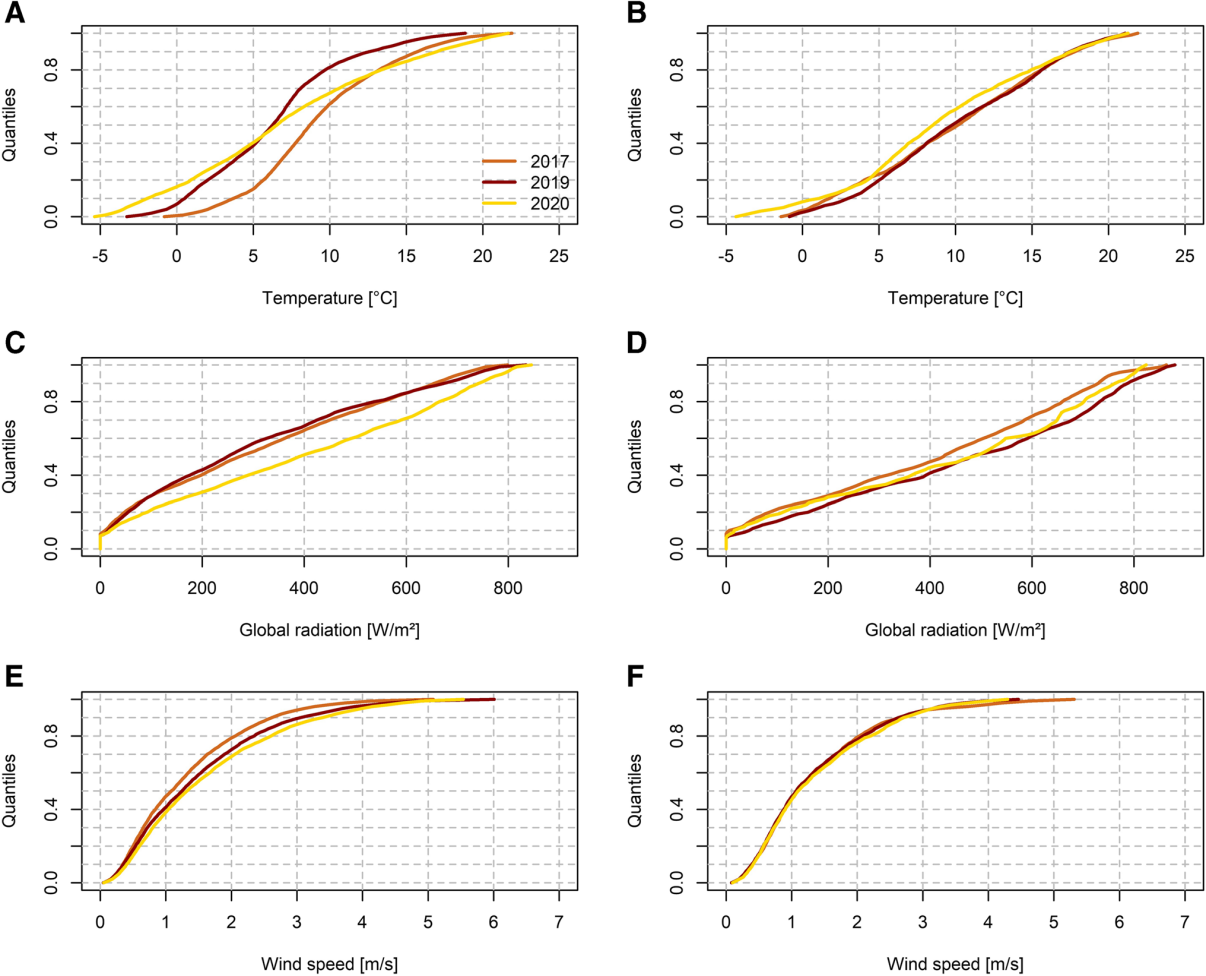
Ecdfs of 2 m air temperature (top), global radiation (middle) and wind speed (bottom) for the sector W in 2017, 2019 and 2020 (colour coded). Left column (**A**, **C**, **E**) shows data prior to application of the MFM, right column (**B**, **D**, **F**) shows data after application of the MFM

To analyse whether the meteorological filtering method (MFM) performs satisfactorily and thus provides a meaningful sample of episodic data (i.e. in our case for the ISDP20 with the same time period during 2017 and 2019), we calculate the empirical cumulative distribution functions (ecdf) derived from hourly 2 m air temperature, global radiation and wind speed of individual measurement sites for both meteorological sample days and all days within the specified periods, and compare their sector-specific averages. In Fig. 2, we illustrate this comparison exemplarily for sector W (figures for other domains are provided in the supplement, see Figs. S1-3). This figure shows that the MFM performs predominantly well (i.e. harmonises the ecdfs of the selected variables). To quantify the agreement between the individual samples, we calculate the root mean square error (RMSE) of the average ecdfs relative to 2020 values. A comparison for subdomain RMSE values per variable and comparison period is given in Table 1. Here, it can be clearly seen that the RMSE is strongly reduced for the MFM sample (about a factor of ~2–3), confirming the general assumption of an improved comparability of the data after application of the MFM. Thus, the application of the MFM allows for an analysis of changes in pollutant concentrations relatable to alterations in direct emissions or emissions of pollutant precursors without neglecting effects of meteorological variability. This benefit, however, comes at the cost of a limitation of the overall sample size.

**Fig. 3:**
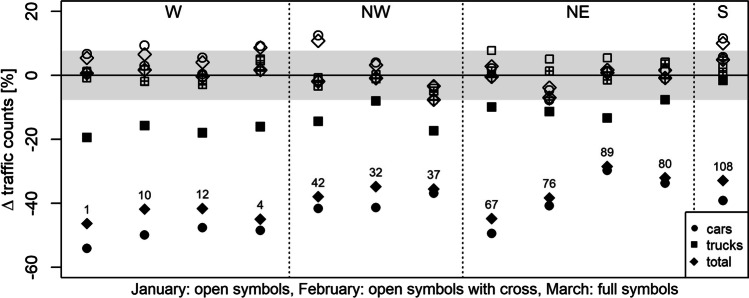
Relative traffic count differences between 2020 and 2019 from ASFiNAG data. Grey shading marks the range within which changes are considered as non-significant. Individual columns (numbered above the second symbol from below) refer to the air quality monitoring site in closest proximity to the traffic monitor (for details (location, sector, classification type and area as well as pollutants monitored) see supplemental Table S1)

**Table 1:** RMSE values of 2 m temperature, global radiation and wind velocity ecdfs for the comparison of MFM and full samples (given in parentheses) of 2020 relative to 2017 (top) and 2019 (bottom). Bold numbers highlight the lower RMSE value comparing the MFM and full samples per sector, variable and year

Sector/variable	2 m temperature	Global radiation	Wind velocity
	RMSE 2017		
W	**1.2** (3.2) **1.7** (3.2) **1.9** (3.6) **0.6** (4.3)	**42.0** (95.1) **71.5** (124.8) **48.8** (105.0) **17.5** (50.6)	**0.2** (0.4) 0.3 (0.3) **0.1** (0.6) **0.2** (0.3)
NW			
NE			
S			
	RMSE 2019		
W	**1.4** (2.4) **1.5** (2.4) **2.3** (2.8) **1.7** (3.0)	**30.0** (108.9) **18.0** (115.5) **28.7** (94.2) **35.7** (110.3)	**0.1** (0.2) 0.9 (**0.2**) **0.4** (0.8) 0.4 (**0.1**)
NW			
NE			
S			

### NO_x_ concentration changes during the ISDP20

During the ISDP20, traffic volume was substantially reduced in Austria, but not uniformly in all regions (see Fig. 3). Individual mobility (passenger cars) during March 2020 has decreased roughly between −60 and −30% compared to 2019, which coincides well with traffic reductions observed in other European countries (e.g. Higham et al. 2021; Sbai et al. 2021; Spohn et al. 2022). The largest reductions (about −50 to −60% for individual traffic) have been observed in the sector W along the much-frequented motorways (Inn valley, Brenner) connecting Germany with Italy. Compared to passenger traffic, heavy duty traffic (trucks) has been reduced to a far less extent (up to −20%) and quite uniformly in all regions in Austria during ISDP20.

**Fig. 4:**
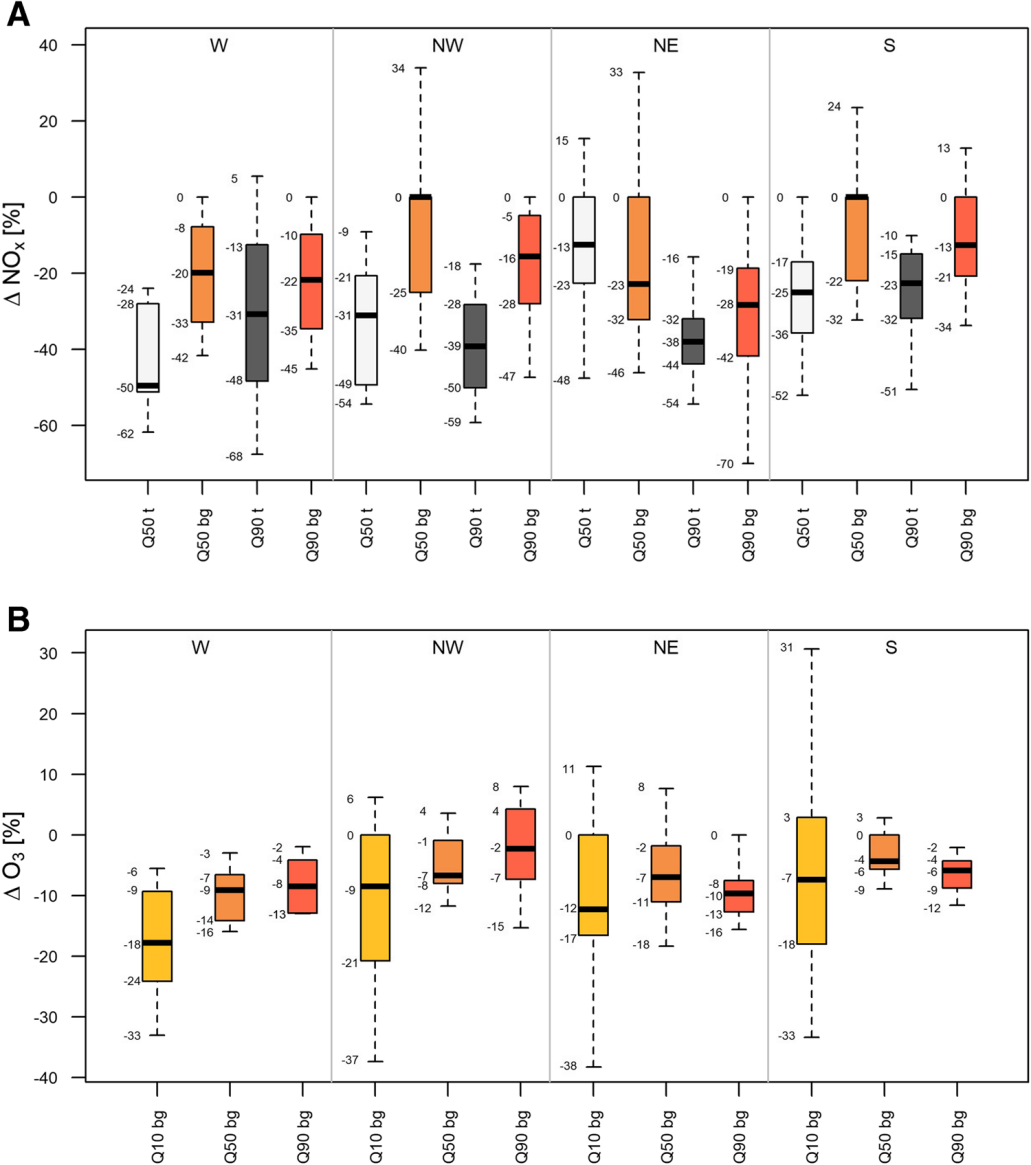
Relative changes in Q50 and Q90 of NO_x_ (**A**) and Q10, Q50 and Q90 of O_3_ (**B**), per sector and site classification (traffic (t; grey-scale) vs. background (bg; coloured), each category (t and bg) comprising rural, suburban and urban sites). All data corresponds to MFM filtered data. Note, outliers (16 data points in total, exceeding 1.5 times the interquartile range) in the half-hourly data have been omitted

To investigate the effect of reduced road traffic on ambient NO_x_ levels during the ISDP20, we turn the focus first on traffic sites (Fig. 4A) because changes in traffic volume are assumed to translate directly into NO_x_ concentrations. A view on sector W confirms this assumption. Here all rural monitors are traffic sites, located next to the various highways, and the quantified NO_x_ reductions of −50 and −31% for Q50 and Q90 (see Fig. 4A), respectively, match closely the observed reduction in traffic volume (about −45%) shown in Fig. 3.

Expanding the analysis to all NO_x_ monitoring stations (traffic and background) reveals a much more diverse picture, but the majority of sites still show significant NO_x_ reductions. NO_x_ changes at traffic sites range between −62 and +15% for Q50 and −68 and +5% for Q90, respectively. Also, the vast majority of background stations show reduced NO_x_ concentrations during ISDP20, independent of the subdomain classification (see Fig. 4A). At background sites (defined as all sites but traffic sites), NO_x_ changes range between −46 and +34% for Q50 and −70 and +13% for Q90, respectively. The largest NO_x_ reductions at background stations are identified in the NE sector, the regional domain where also Austria’s capital Vienna is located.

**Fig. 5:**
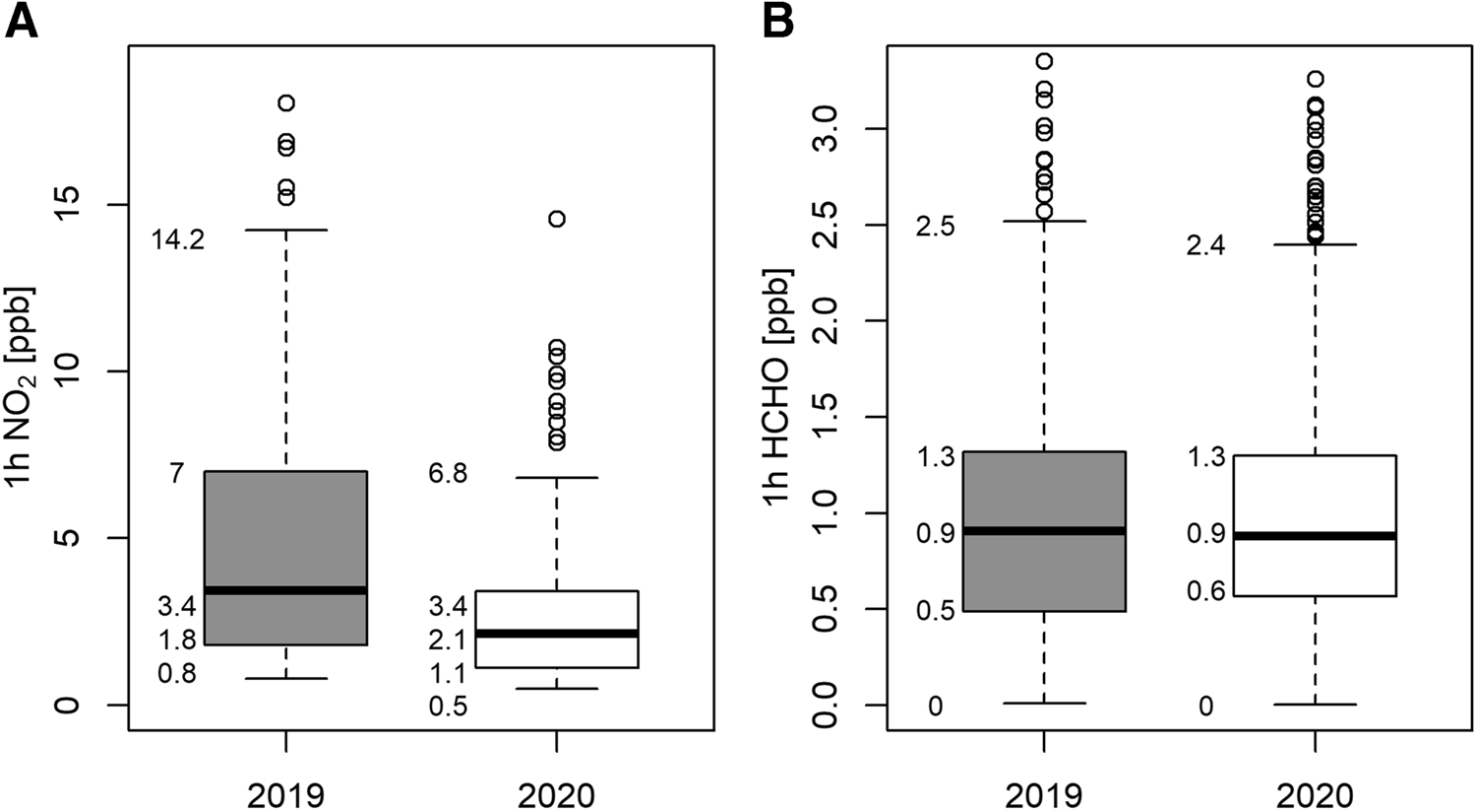
Distribution of 1 h average MFM sampled NO_2_ (**A**) and HCHO mixing ratios (**B**) derived for the MAX-DOAS path over the Vienna city centre during ISDP20 and the comparable time period in 2019

For the Vienna metropolitan region, the results of the station-based analysis can be compared to spatial MAX-DOAS NO_2_ mixing ratios. MFM sampled MAX-DOAS measurements show an NO_2_ reduction of −1.3 ppb (−38%) for Q50 (see Fig. 5A), which is in good agreement with the results detailed above. In addition, these findings coincide well with the results reported by the department of environmental protection of the city of Vienna (MA22 2021) and Brancher (2021). Since the MAX-DOAS instrument measures about 100 m above ground, NO_2_ mixing ratios are expected to be smaller than at the street level. In contrast to NO_2_, MAX-DOAS measurements of HCHO do not indicate a significant change in VOC mixing ratios during ISDP20 compared to the preceding year (see Fig. 5B).

### O_3_ concentration changes during the ISDP20

The Austrian monitoring network comprises only few traffic sites measuring ozone, hence the nationwide analysis of surface ozone changes during ISDP20 relies on background monitors. Compared to 2019, 2020 shows a decrease in concentrations at the “lower tail” (Q10) of the O_3_ distribution. We identify however also locally pronounced increases at some suburban and urban sites (e.g. +50 to +250% in Salzburg), which can be explained by reduced O_3_ titration (note that in Fig. 4B, these outliers are not shown because they lie far beyond the selected plotting range). For the median (Q50) and the “upper tail” (Q90) of the ozone distribution, moderate decreases in ozone concentration are observed at most sites, in magnitude largest for the NE sector. For Q50 and Q90, ozone changes range between −18 to +8% and −16 to +8%, respectively. These substantially smaller changes in ozone, compared to NO_x_ concentrations, indicate that changes in precursor NO_x_ do not translate linearly into O_3_ concentration changes. In addition, the relatively small O_3_ concentration changes along with the similar changes observed across sectors indicate the importance of continental/regional background O_3_ concentration levels and ambient meteorological conditions (particularly the limitation by springtime radiation levels) over NO_x_ or VOC concentrations at that time of the year.

### Changes of the NO_2_, NO and O_3_ diurnal cycle during the ISDP20

**Fig. 6:**
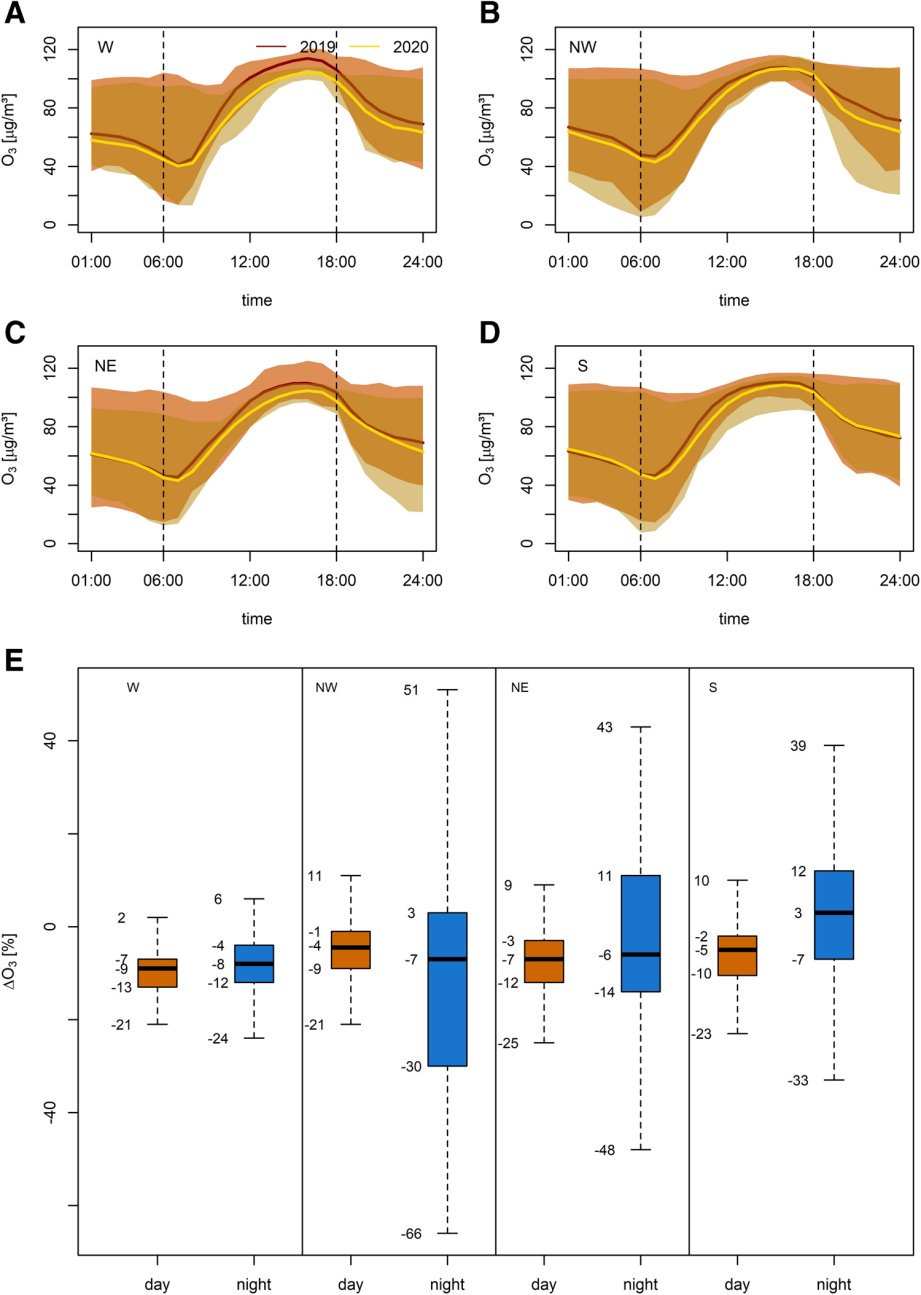
Mean diurnal cycles of O_3_ at background sites (comprising suburban/urban and rural sites) per subdomain: (**A**) W, (**B**) NW, (**C**) NE and (**D**) S. Red (2019) and yellow (2020) shadings indicate the range between maximum and minimum values. Bold lines indicate subdomain averages. Changes in day- (orange) and night-time (blue) variations of mean diurnal cycles relative to 2019 (**E**). Note, outliers (data points exceeding 1.5 times the interquantile range above the upper quartile and below the lower quartile) have been omitted. All data is given as hourly averages

Having explored changes in aggregated pollutant concentrations and probability distributions, we turn the focus to changes in the diurnal cycle, mainly at background sites (see Fig. 6; for a visualisation of suburban/urban and rural measurement sites see supplemental Figs. S6-S7), to identify the impact of NO_x_ concentrations on ambient O_3_ concentrations. In summary, subdomain averages of mean diurnal O_3_ variations show comparably weak changes in response to precursor emission changes. Here, changes relative to 2019 are found between −14 and +3%. In contrast, individual measurement sites reveal stronger variations. Particularly large changes (−66 to +51%) have been identified during night-time, which can be attributed to titration effects. Pronounced night-time changes in O_3_ concentrations have been found across all sectors, with changes in sector W being less pronounced (see Fig. 6E). However, this reduced variability in sector W agrees with the overall reduced variability in O_3_ concentration changes (detailed in Fig. 4B) especially at the lower end of the distribution. As daytime concentrations show only minor variations (−25 to +11%) it is assumed that the sensitivity of ozone production to radiation outweighs changes in precursor NO_x_ during ISDP20.

**Fig. 7:**
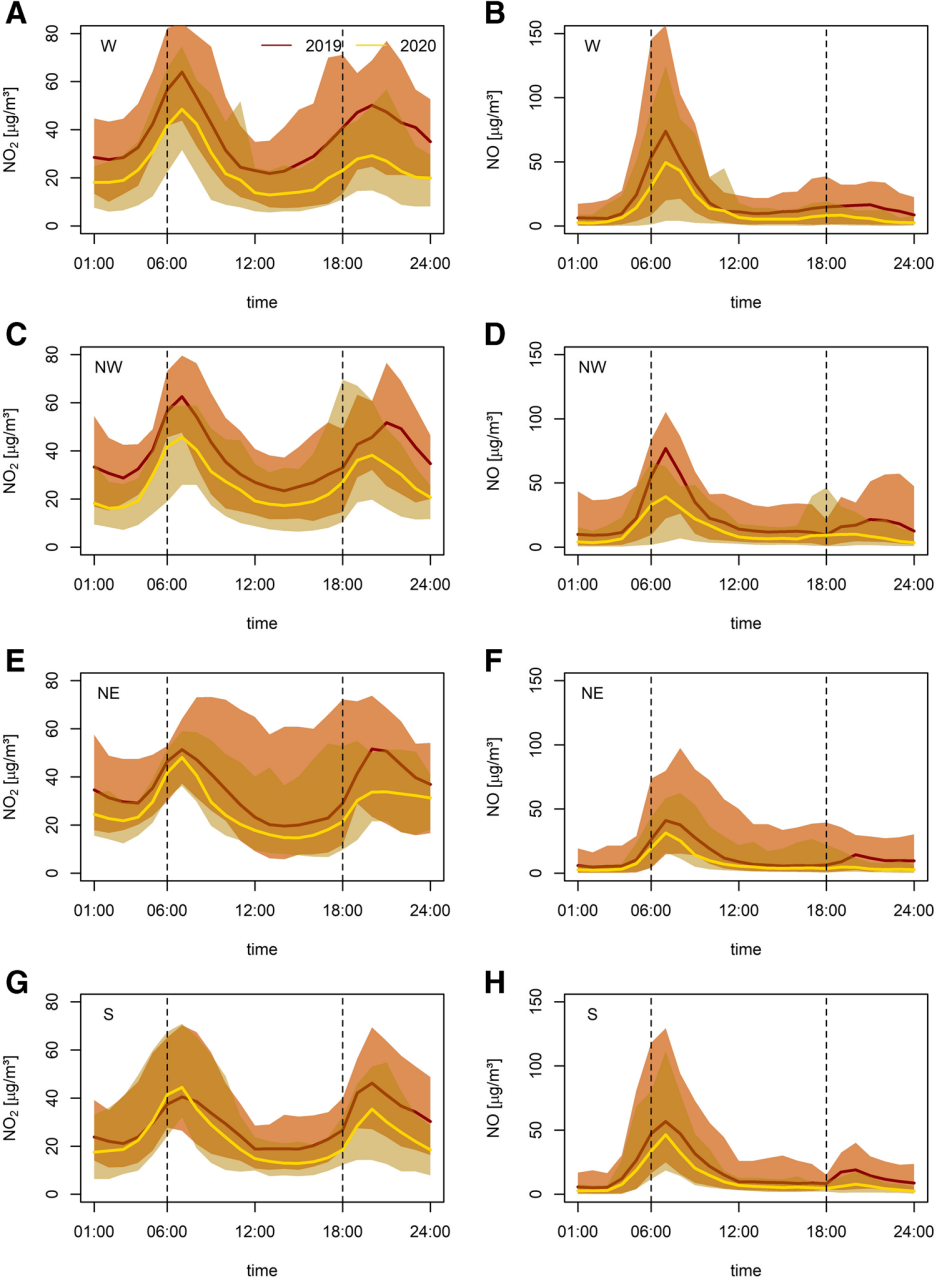
Mean diurnal cycles of NO_2_ (left column) and NO (right column) for traffic sites per subdomain (**A**), (**B**) W, (**C**), (**D**) NW, (**E**), (**F**) NE and (**G**), (**H**) S. Red (2019) and yellow (2020) shadings indicate the range between maximum and minimum values. Bold lines indicate subdomain averages. All data is given as hourly averages

The diurnal cycles of NO_2_ and NO have been modified through emission changes during ISDP20, with largest effects at traffic sites (see Fig. 7). Here subdomain average NO_2_ changes relative to 2019 are predominantly negative (see Fig. 7 left column) with values between −50 and +11% (note slightly higher NO_2_ concentrations in 2020 in the morning in the S domain). For individual traffic monitoring stations, changes in the diurnal variation are even more pronounced, ranging between −72 and +21%. Average NO_2_ changes are slightly smaller at background stations in urban/suburban and rural areas ranging from −49 to +44% and −43 to −17%, respectively (see Figs. S4-5). The magnitude of measurement site-specific decreases is in general comparable to traffic stations. Increases, however, are found to be more distinct, especially in the S subdomain. Here, values vary between −77 and +64% in suburban/urban and −75 to +72% in rural areas. Overall, our analysis reveals larger changes in the daily cycles for NO during ISDP20 than for NO_2_, which we attribute predominantly to fast transition of NO through titration. Here, sector averages of changes at traffic sites range from −75 to −17%, while rural and suburban/urban background stations show changes ranging between −63 to −29% and −77 to +145%, respectively (see Figs. S4-5).

### PM_10_ concentration changes during the ISDP20

Having discussed changes in gaseous pollutants, we turn the focus now to changing PM_10_ concentrations during ISDP20. Our working hypothesis is that PM_10_ levels have been less affected by the decrease in traffic volume during the lockdown than NO_x_ concentrations, as a large fraction of the Austrian PM_10_ pollution is attributable to long-range transport and non-traffic sources such as domestic heating (note: traffic PM emissions account on average only for about 16% of total PM_10_ in Austria) (Anderl et al. 2021). Also, complex PM_10_ emission baskets and an overall decline in concentrations (Spangl and Nagl 2020) complicate a site-level effect analysis of ISDP20 on the ambient PM_10_ concentration.

As the impact of all other PM_10_ emission sources except for traffic and gas-to-particle conversion from precursor NO_x_ are assumed to be widely unaffected by restrictions during ISDP20, we choose to compare in each sector a characteristic background site (marked with a “*” in supplemental Table S1) with eight traffic-influenced sites (marked with a “†” in supplemental Table S1) during lockdown (same time period as ISDP20) and the pre-lockdown period defined as 15.01.–29.02. (weekday only sample).

**Fig. 8:**
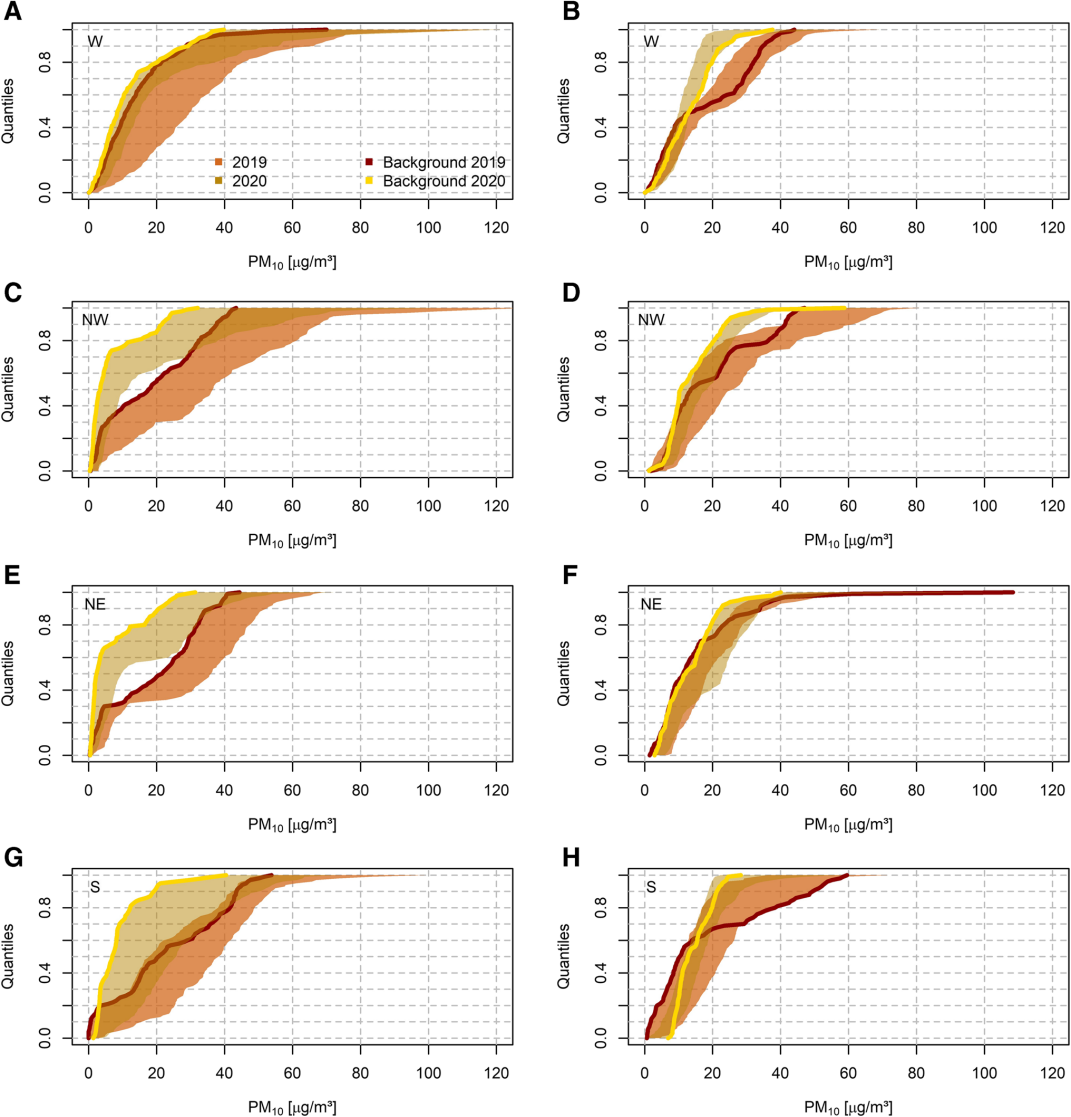
PM_10_ ecdfs for the pre-lockdown (left column) and lockdown (right column) time intervals in 2019 and 2020 per regional domain: (**A**), (**B**) W, (**C**), (**D**) NW, (**E**), (**F**) NE, (**G**), (**H**) S. The coloured shadings (red 2019, yellow 2020) indicate the range of individual quantiles (Qx, calculated as maximum/minimum of selected stations marked with † (traffic influenced) or * (background) in supplemental Table S1), thick lines (red 2019, yellow 2020) show the ecdfs of the domain background reference station

In Fig. 8, we contrast the PM_10_ ecdfs for these periods for the selected background and traffic-influenced sites. For the majority of the sectors, the individual distributions become narrower during ISDP20, and especially at the upper tail decreasing concentrations are observed. We summarise this exemplarily for the Q50 and Q90 values (calculated as absolute difference between the respective maximum and minimum values) in the supplemental Table S4. In this regard, 2019 ecdf bandwidths during the lockdown period are spanning from 6.8 to 12.9 μg/m^3^ (Q50) and 10.2 to 28.3 μg/m^3^ (Q90). In contrast, ISDP20 bandwidths are ranging from 3.4 to 10.9 μg/m^3^ for the median and 6.9 to 11.6 μg/m^3^ for the “upper tail” of the distribution, respectively. Absolute PM_10_ differences are predominantly larger during both pre-lockdown and lockdown periods in 2019 compared to 2020. In 2020, we find however also an overall shift of traffic site PM_10_ concentrations towards the concentration regime of typical background sites. Hence, we identify also a significant influence of ISDP20 measures on PM_10_ via reduced road emissions.

## Summary, discussion & conclusions

Emission changes following COVID-19 related restrictions have been closely examined by the scientific community, and over the last months, several studies have been published documenting the impact of lockdown measures on local/regional/global air quality. Here, we add to the efforts of COVID-19 related air quality research by analysing changes in pollutant levels during ISDP20 in Austria while accounting for ambient meteorological conditions through the application of an MFM. The MFM has the advantage that it allows us to analyse changes in air pollutant concentrations attributable to changes in direct emissions or emissions of pollutant precursors without neglecting effects of meteorological variability. The advantage of the MFM comes, however, at the expense of limiting the overall sample size available for comparison of ambient air quality during ISDP20 with those in neighbouring years and does not allow us to fully compare conditions during ISDP20 with those in directly preceding months.

Our analysis shows that the largest relative concentration changes among air pollutants considered are found for NO_x_, with particularly large changes in the upper tail of the concentration distribution, varying between −68 and +5% at traffic sites. These drastic changes in NO_x_ concentrations reflect the substantial decrease in private and commercial transportation during ISDP20. Background monitoring sites show also large NO_x_ reductions, but less pronounced than for traffic sites. Overall, our findings agree broadly with other recent studies examining NO_x_ changes during spring 2020 in Europe (e.g. Deroubaix et al. 2020; Higham et al. 2021; Sbai et al. 2021; Shi et al. 2021).

The ratio of NO_x_ to VOC concentrations determines the chemical regime for maximum O_3_ production (i.e. NO_x_ or VOC limitation). Commonly NO_x_ emission reductions are expected to translate into lower peak O_3_ concentrations in NO_x_ limited regimes (this is usually the case in rural areas), whereas no substantial changes are anticipated for VOC-limited areas (urban areas). This assumption is valid if sufficient solar radiation is available. At mid-latitudes, this premise is not fulfilled in early spring (March, April) when the observed daily global radiation sum ranges approximately between 4.5 and 6 kW/m^2^ on clear sky days. Since observed peak O_3_ production is substantially constrained by radiation abundance, the difference in O_3_ between NO_x_- and VOC-limited regimes is assumed to be small and lockdown-related NO_x_ reductions are expected to impact ozone concentrations to a much smaller extent. In contrast titration is assumed to have a greater impact.

Following the significant decrease of NO_x_ concentrations, propagating effects for surface O_3_ levels are expected. Changes in the O_3_ concentrations at background sites show a rather weak (but predominantly decreasing) response to NO_x_ declines varying between roughly −18 to +8% for both the median and the upper tail of the distribution. The analysis of areal mean HCHO mixing ratios available from the Vienna MAX-DOAS network does not show a significant difference between 2019 and 2020 and hence provides confidence that the observed O_3_ variations at suburban/urban monitors are predominantly attributable to NO_x_ changes. Thus, we conclude that NO_x_ reductions would have had a larger effect on surface O_3_ if the lockdown had not occurred during spring but summer when radiation and hence photochemical O_3_ production is at its maximum. While the analysis of subdomain averages of diurnal cycles of both NO_x_ and O_3_ show reduced pollution levels during ISDP20, examination of individual site records reveals large night time O_3_ variability, resulting from altered titration. However, double digit increases in O_3_ concentrations as found in other studies (e.g. Deroubaix et al. 2020; Grange et al. 2021; Ordóñez et al. 2020; Sbai et al. 2021; Sicard et al. 2020a) cannot be identified in our study region.

PM_10_ concentrations have been declining in Austria during recent years and this trend has continued in 2020 (Anderl et al. 2021; Spangl 2021; Spangl and Nagl 2020). Our analysis focusing on ISDP20 highlights a pronounced decline in the width of the upper tail of the PM_10_ distributions and a convergence of concentrations at traffic sites towards background levels. We find that the magnitude of PM_10_ changes varies strongly between individual sectors with strongest changes in the S subdomain and weakest changes in the NE sector. For the NE sector, we also found increases around the median of the distribution for multiple sites compared to the reference year which is not found in the other subdomains.

Overall, our study highlights the importance of considering ambient meteorology when evaluating changes in ambient pollution levels and illustrates that changes in pollutant burdens during ISDP20 are comparable to results reported in other European countries and regions. We see overall the largest impact of lockdown measures on concentrations of NO_2_ and NO, followed by particulates in traffic settings. Ozone concentrations appear, when considering MFM sampled data, less affected given the limited photochemical potential during the seasonal timing of the lockdown. Nevertheless, the effects of reduced NO_x_ burdens emerge in O_3_ daily cycles through effects of precursor limitation at day and reduced night time titration. Air quality changes during ISDP20 show the effect of marked temporary emission reductions. The analysis of potential long-term (all-season) or continued (multi-year) effects of sustained emission reductions are beyond the scope of the present observation-based study but intended for modelling studies in future work.

## Supplementary information

Fig. S1 Ecdfs of key meteorological variables during ISDP20 and corresponding time intervals in 2017 and 2019 for sector NW prior to application of the MFM (A, C, E) and after application (B, D, F). (PNG 64 kb)

High Resolution Image (TIFF 232 kb)

Fig. S2 Ecdfs of key meteorological variables during ISDP20 and corresponding time intervals in 2017 and 2019 for sector NE prior to application of the MFM (A, C, E) and after application (B, D, F). (PNG 64 kb)

High Resolution Image (TIFF 233 kb)

Fig. S3 Ecdfs of key meteorological variables during ISDP20 and corresponding time intervals in 2017 and 2019 for sector S prior to application of the MFM (A, C, E) and after application (B, D, F). (PNG 63 kb)

High Resolution Image (TIFF 231 kb)

Fig. S4 Mean daily cycles of NO_2_ for individual sectors (W (A – C), NW (D – F), NE (G – I) & S (J – L)) averaged by station type (traffic (left column), suburban/urban background (centre column) & rural background (right column)). Red (2019) and yellow (2020) shadings indicate the range between maximum and minimum values. Bold lines indicate subdomain averages. All data is given as hourly averages. Note, missing shading in panel (B) is due to insufficient number of measurement sites. Y-axis range differs among panels in the right column. (PNG 121 kb)

High Resolution Image (TIFF 482 kb)

Fig. S5 Mean daily cycles of NO for individual sectors (W (A – C), NW (D – F), NE (G – I) & S (J – L)) averaged by station type (traffic (left column), suburban/urban background (centre column) & rural background (right column)). Red (2019) and yellow (2020) shadings indicate the range between maximum and minimum values. Bold lines indicate subdomain averages. All data is given as hourly averages. Note, missing shading in panel (B) is due to insufficient number of measurement sites. Y-axis range differs among panels in the right column. (PNG 104 kb)

High Resolution Image (TIFF 429 kb)

Fig. S6 Mean daily cycles of O_3_ at suburban/urban background monitoring sites for individual sectors (W (A), NW (B), NE (C) & S (D)). Red (2019) and yellow (2020) shadings indicate the range between maximum and minimum values. Bold lines indicate subdomain averages. Changes in day- (orange) and night-time (blue) variations of mean diurnal cycles relative to 2019 (E). Note, outliers (data points exceeding 1.5 times the interquartile range) have been omitted. All data is given as hourly averages. (PNG 69 kb)

High Resolution Image (TIFF 300 kb)

Fig. S7 Mean daily cycles of O_3_ at rural background monitoring sites for individual sectors (W (A), NW (B), NE (C) & S (D)). Red (2019) and yellow (2020) shadings indicate the range between maximum and minimum values. Bold lines indicate subdomain averages. Changes in day- (orange) and night-time (blue) variations of mean diurnal cycles relative to 2019 (E.). Note, outliers (data points exceeding 1.5 times the interquartile range) have been omitted. All data is given as hourly averages. (PNG 64 kb)

High Resolution Image (TIFF 280 kb)

Table S1 (DOCX 37 kb)

Table S2 (DOCX 14 kb)

Table S3 (DOCX 14 kb)

Table S4 (DOCX 14 kb)

## Data Availability

The data sets analysed during the current study are not publicly available as ownership rights lie with ZAMG and the Austrian Environment Agency, but are available from the corresponding author upon reasonable request.
