# Supplementary figures and images for: Quantifying changes in ambient NOx, O3 and PM10 concentrations in Austria during the COVID-19 related lockdown in spring 2020
Source: Air Qual Atmos Health. 2022 Jul 22;15(11):1993–2007. doi: 10.1007/s11869-022-01232-w (PMC9305063; doi:10.1007/s11869-022-01232-w)

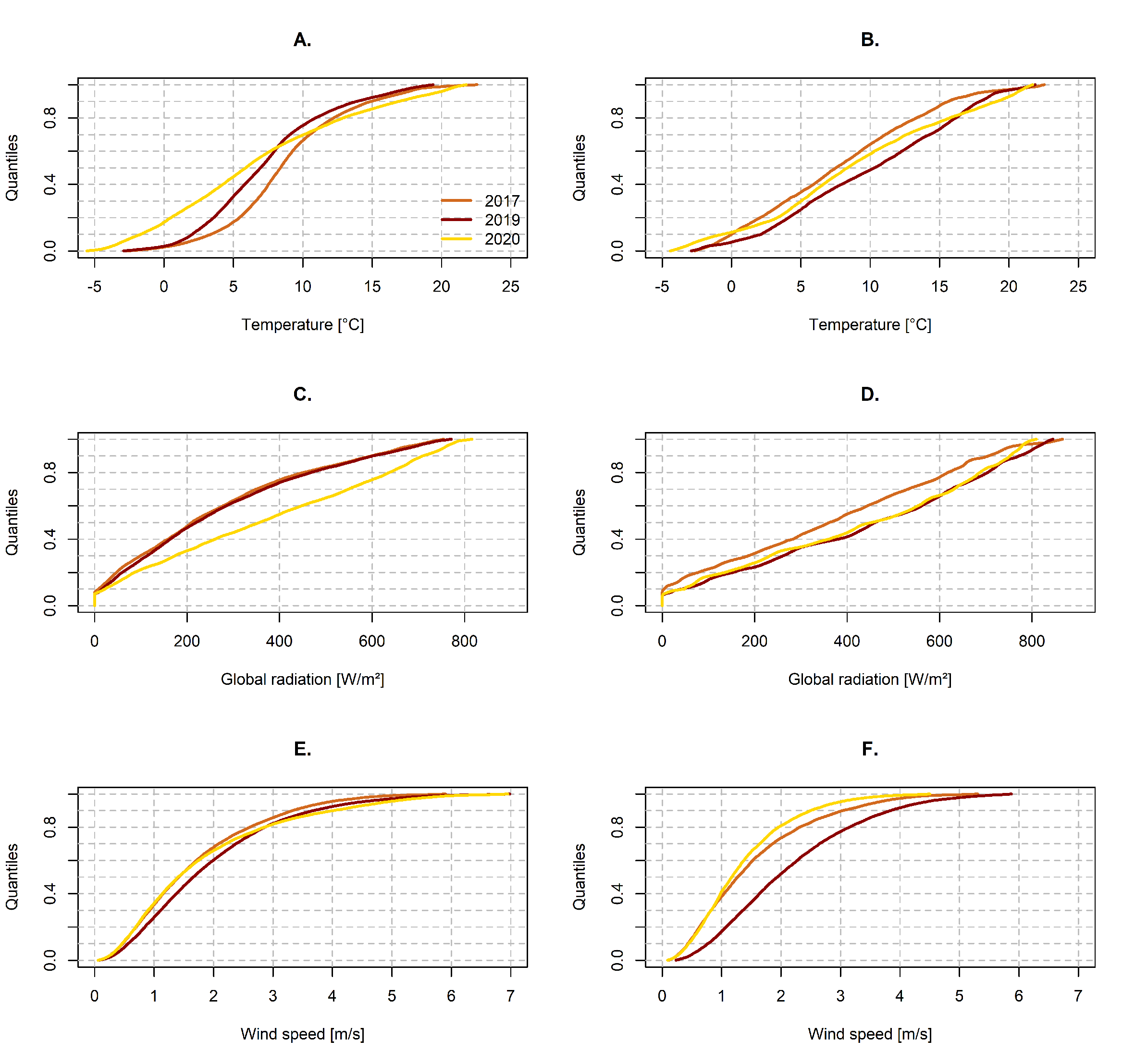

Supplement: Supplementary file 1 — Ecdfs of key meteorological variables during ISDP20 and corresponding time intervals in 2017 and 2019 for sector NW prior to application of the MFM (A, C, E) and after application (B, D, F). (PNG 64 kb) [file 11869_2022_1232_Fig9_ESM.png]

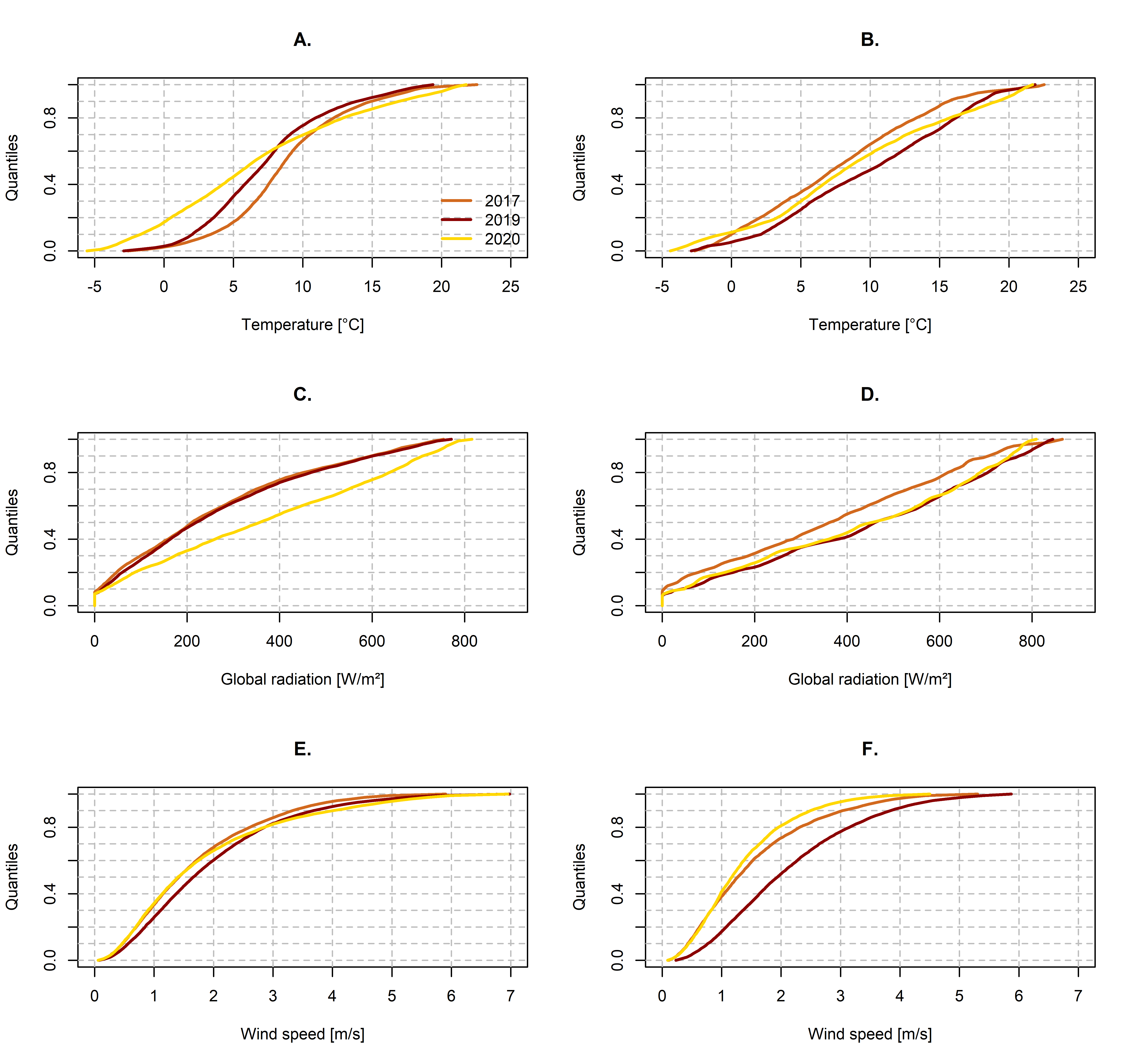

Supplement: Supplementary file 2 — High Resolution Image (TIFF 232 kb) [file 11869_2022_1232_MOESM1_ESM.tiff]

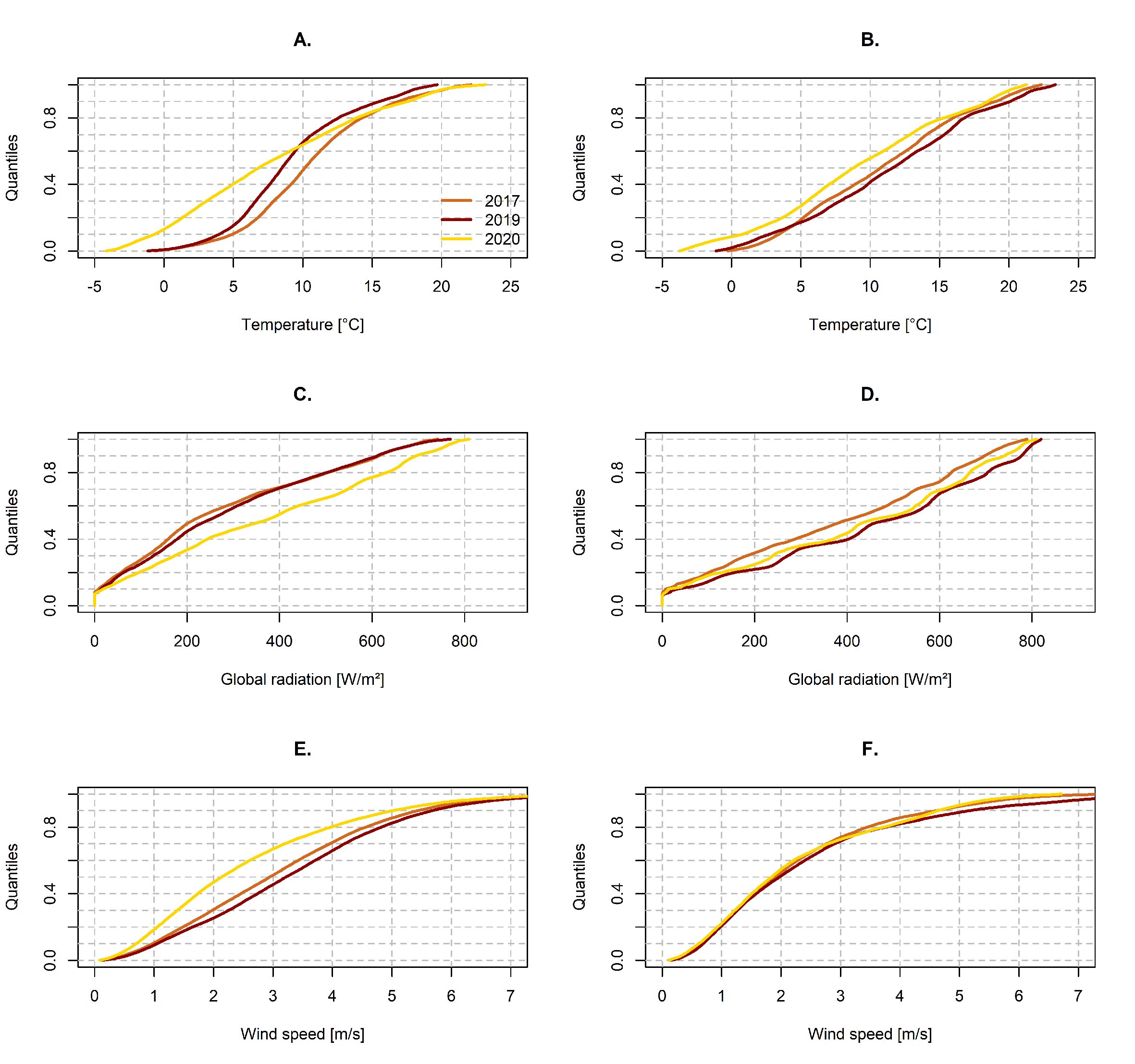

Supplement: Supplementary file 3 — Ecdfs of key meteorological variables during ISDP20 and corresponding time intervals in 2017 and 2019 for sector NE prior to application of the MFM (A, C, E) and after application (B, D, F). (PNG 64 kb) [file 11869_2022_1232_Fig10_ESM.png]

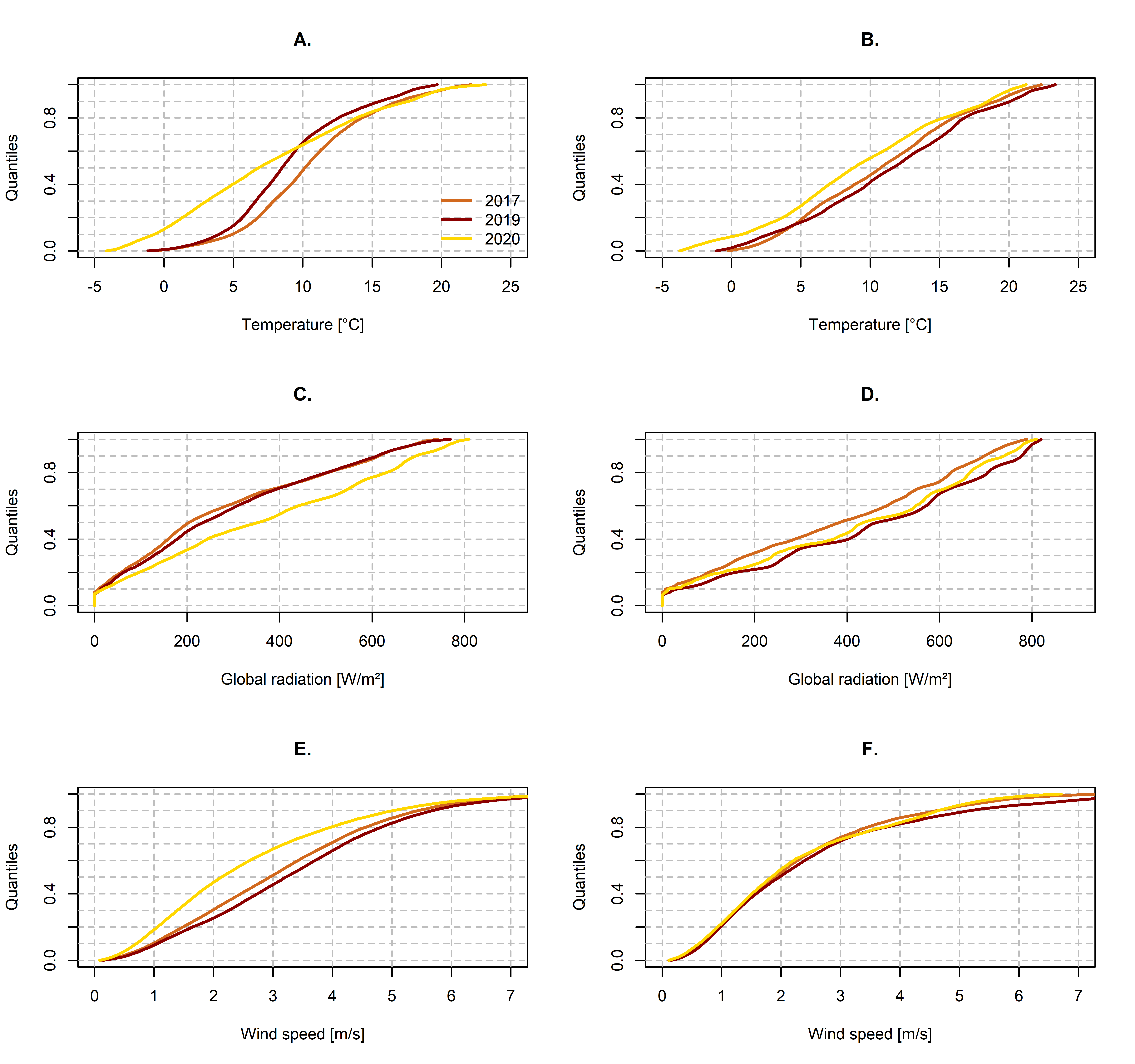

Supplement: Supplementary file 4 — High Resolution Image (TIFF 233 kb) [file 11869_2022_1232_MOESM2_ESM.tiff]

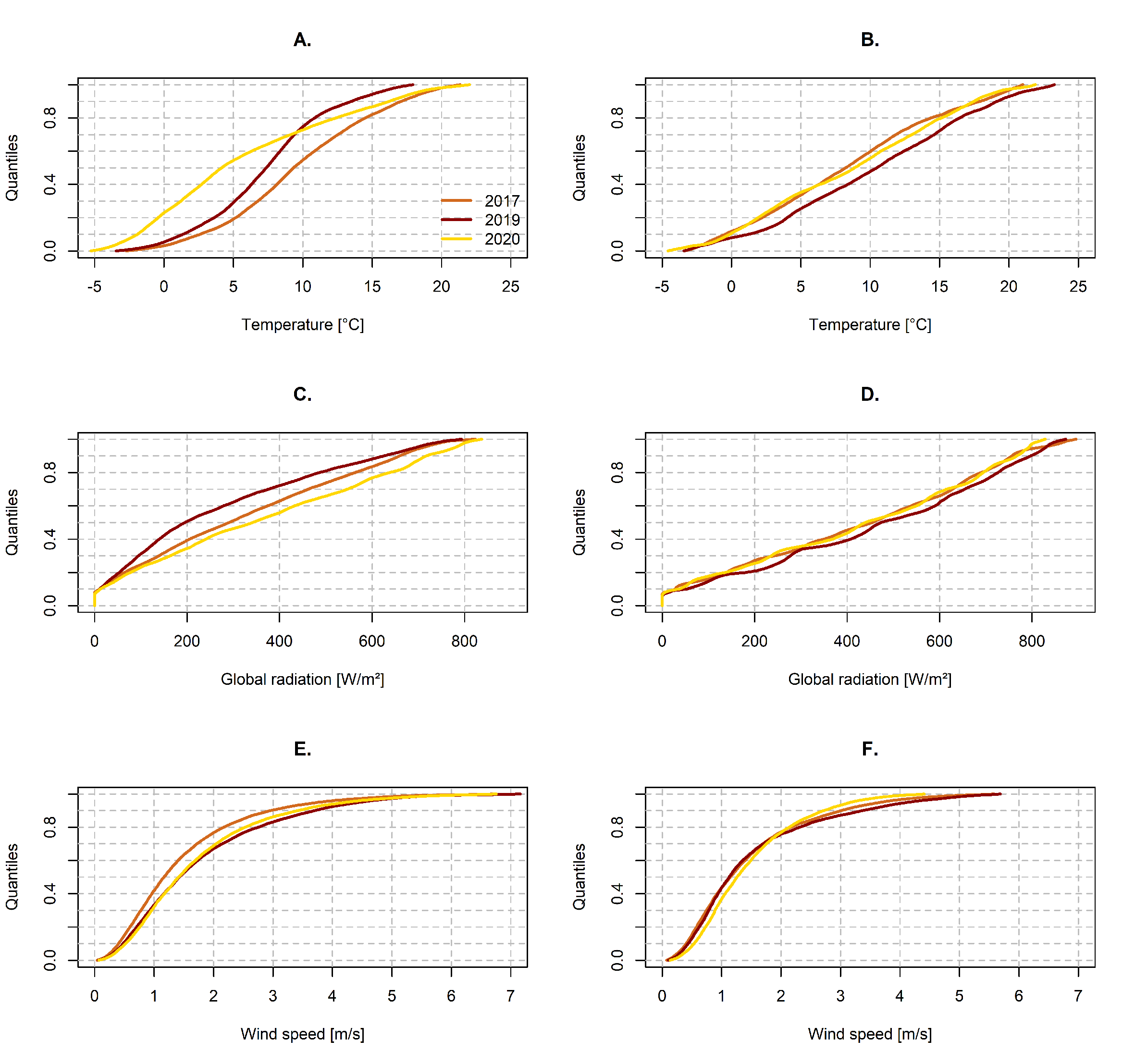

Supplement: Supplementary file 5 — Ecdfs of key meteorological variables during ISDP20 and corresponding time intervals in 2017 and 2019 for sector S prior to application of the MFM (A, C, E) and after application (B, D, F). (PNG 63 kb) [file 11869_2022_1232_Fig11_ESM.png]

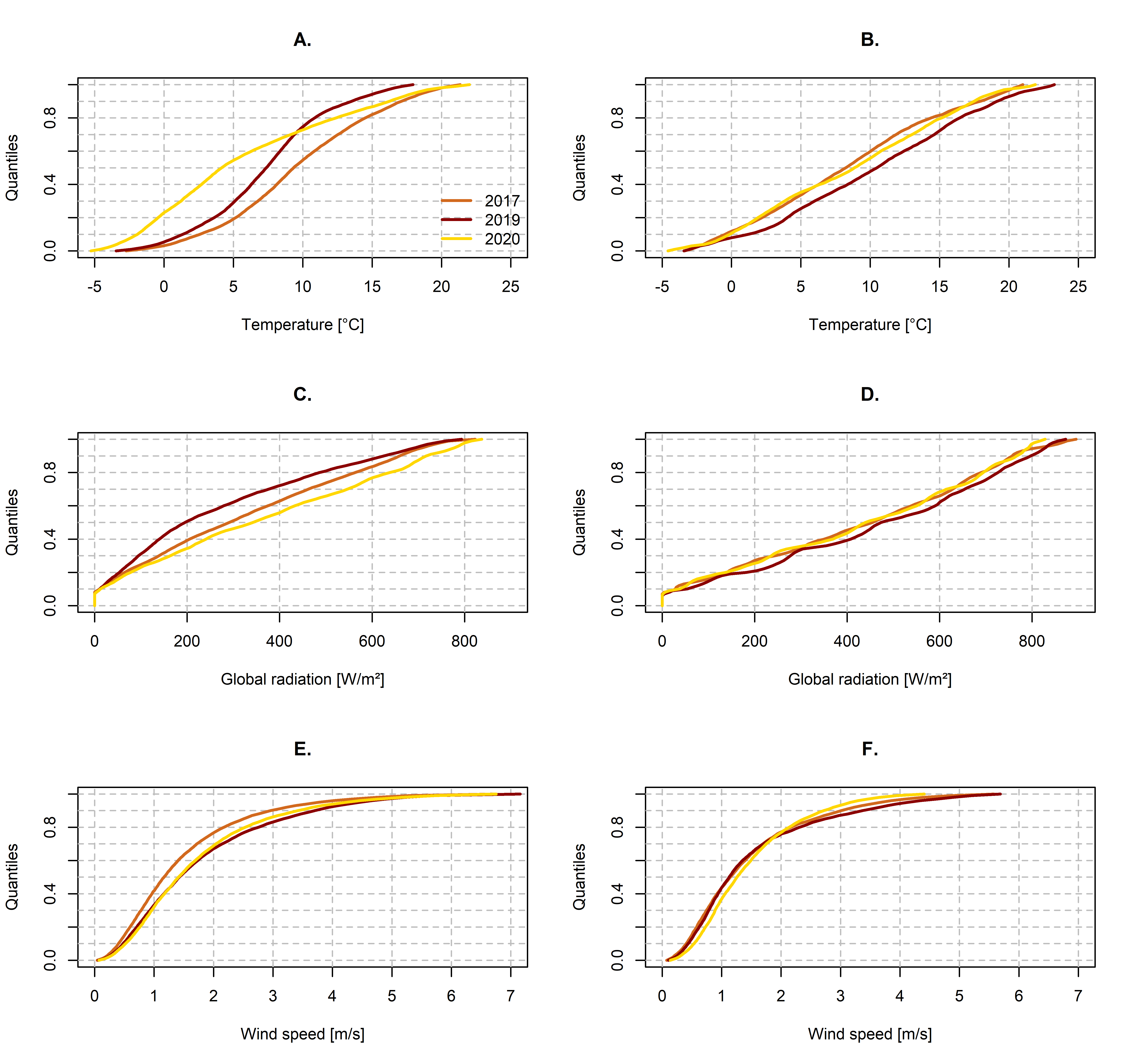

Supplement: Supplementary file 6 — High Resolution Image (TIFF 231 kb) [file 11869_2022_1232_MOESM3_ESM.tiff]

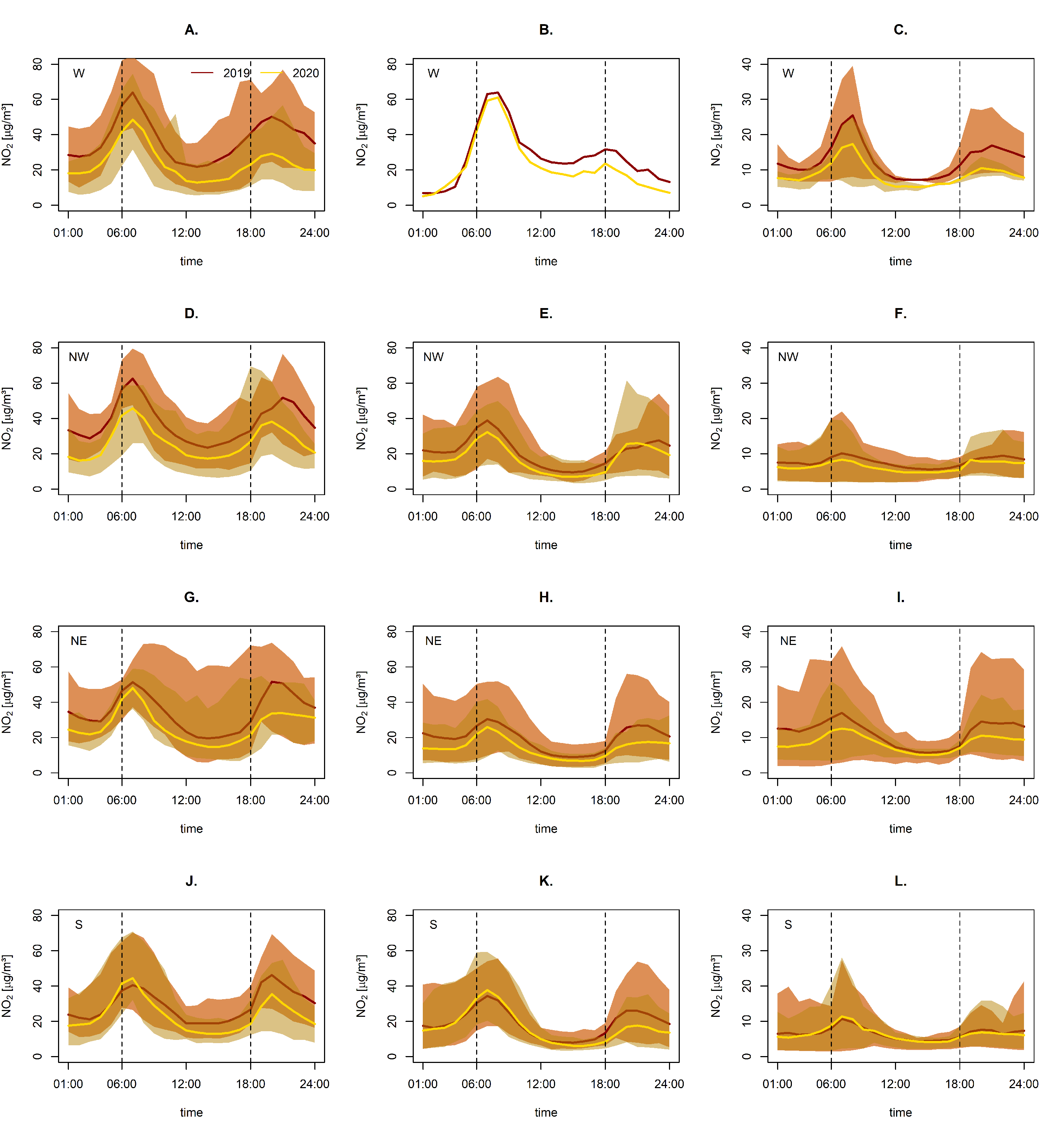

Supplement: Supplementary file 7 — Mean daily cycles of NO2 for individual sectors (W (A – C), NW (D – F), NE (G – I) & S (J – L)) averaged by station type (traffic (left column), suburban/urban background (centre column) & rural background (right column)). Red (2019) and yellow (2020) shadings indicate the range between maximum and minimum values. Bold lines indicate subdomain averages. All data is given as hourly averages. Note, missing shading in panel (B) is due to insufficient number of measurement sites. Y-axis range differs among panels in the right column. (PNG 121 kb) [file 11869_2022_1232_Fig12_ESM.png]

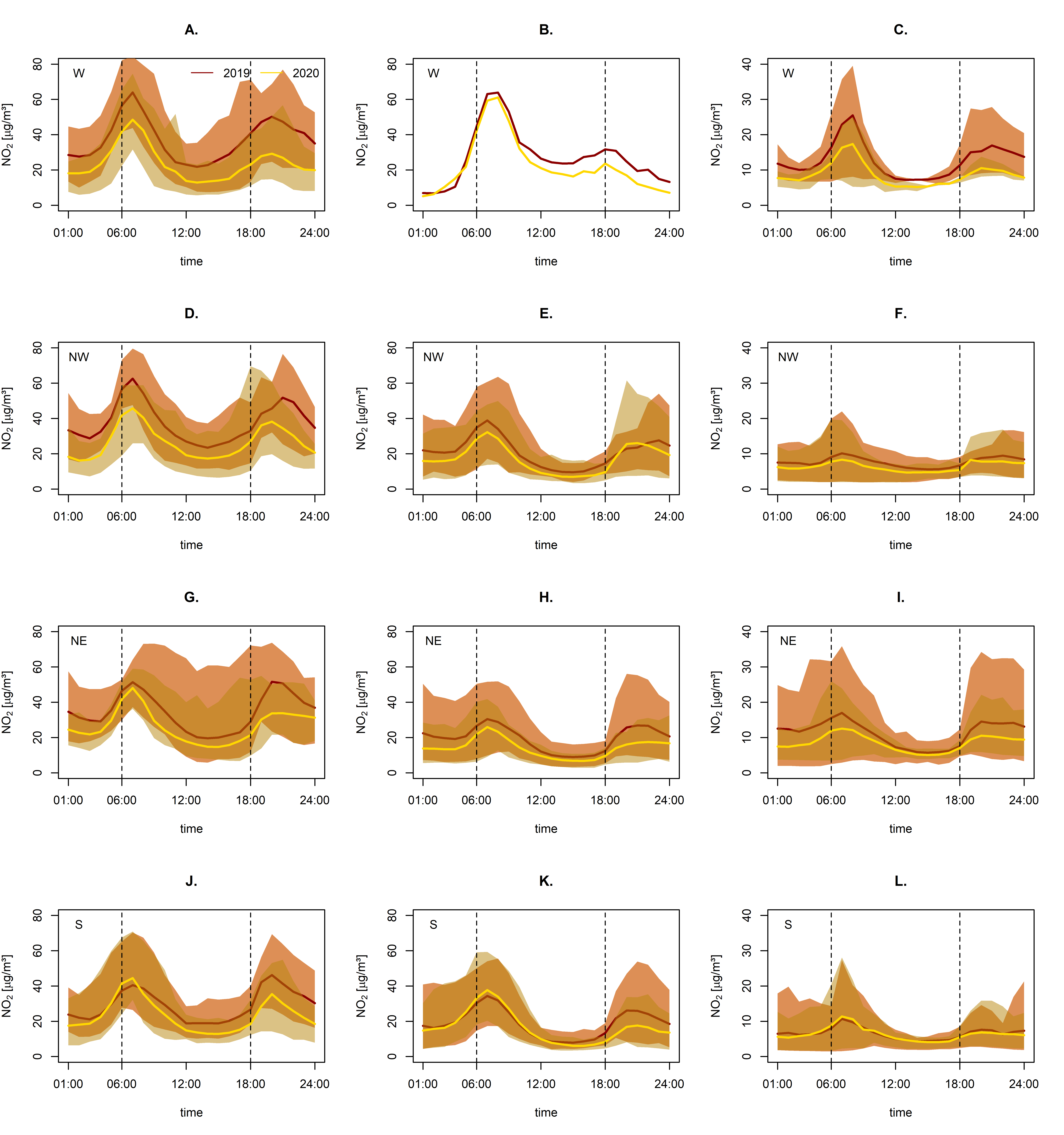

Supplement: Supplementary file 8 — High Resolution Image (TIFF 482 kb) [file 11869_2022_1232_MOESM4_ESM.tiff]

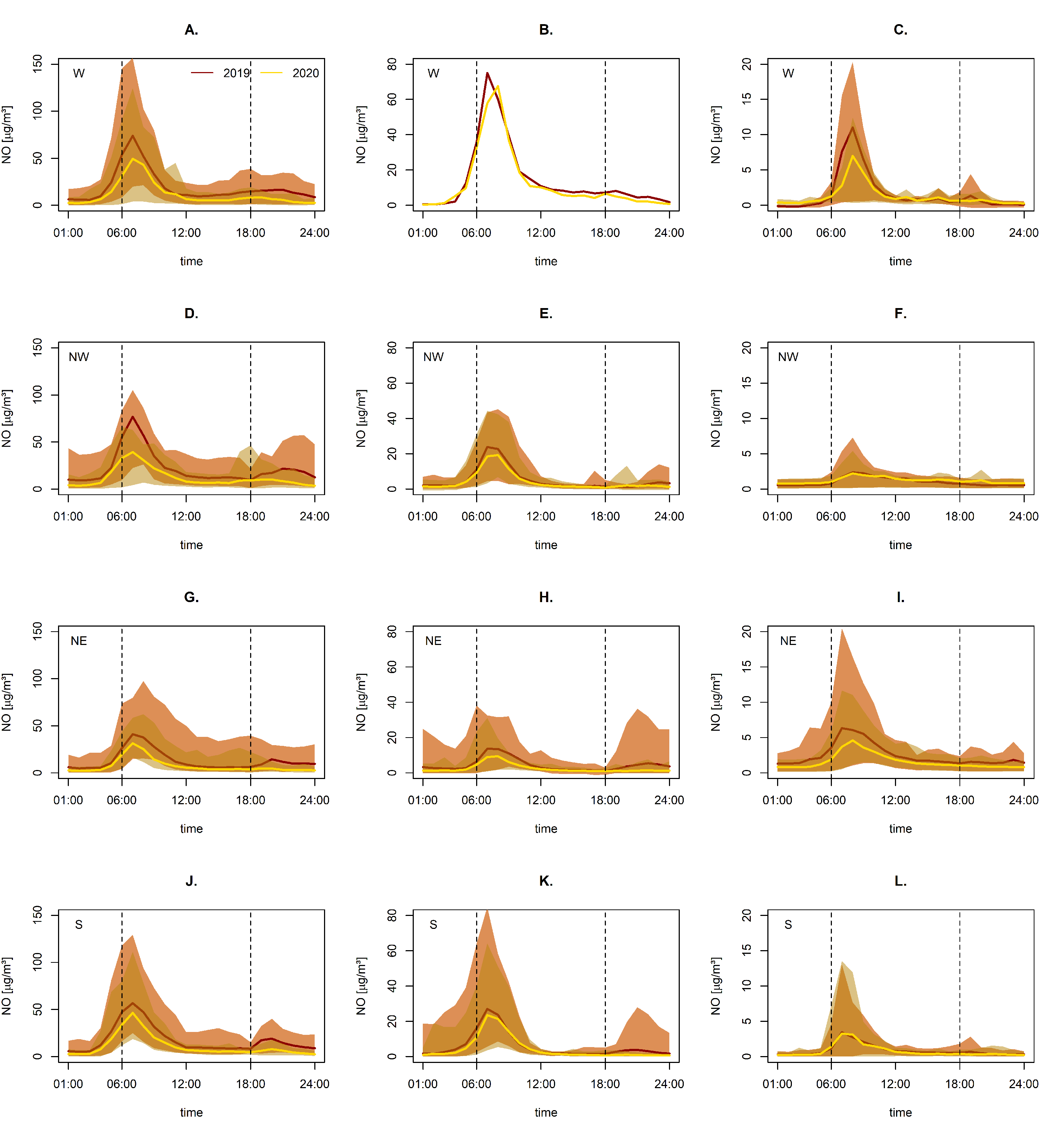

Supplement: Supplementary file 9 — Mean daily cycles of NO for individual sectors (W (A – C), NW (D – F), NE (G – I) & S (J – L)) averaged by station type (traffic (left column), suburban/urban background (centre column) & rural background (right column)). Red (2019) and yellow (2020) shadings indicate the range between maximum and minimum values. Bold lines indicate subdomain averages. All data is given as hourly averages. Note, missing shading in panel (B) is due to insufficient number of measurement sites. Y-axis range differs among panels in the right column. (PNG 104 kb) [file 11869_2022_1232_Fig13_ESM.png]

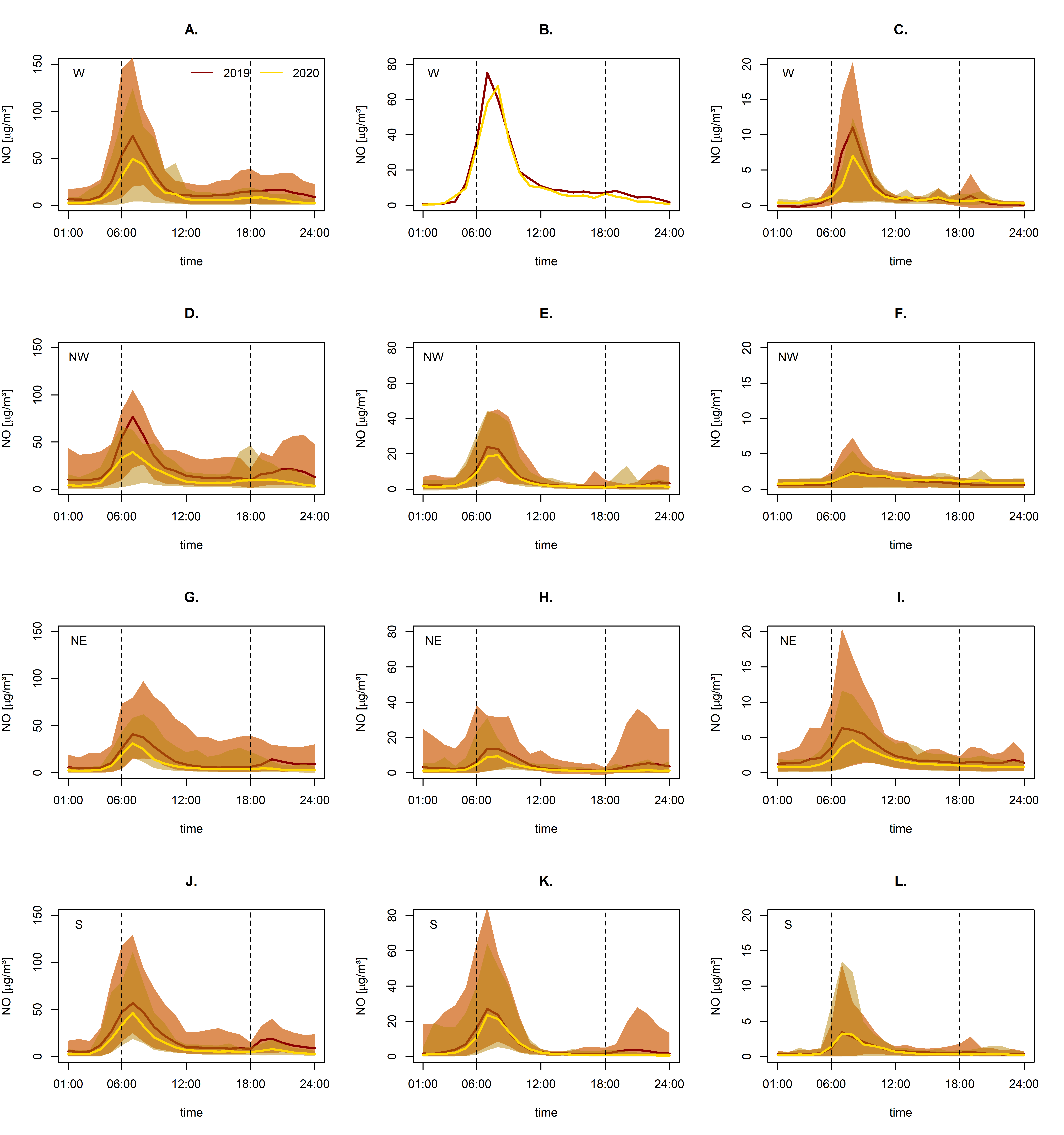

Supplement: Supplementary file 10 — High Resolution Image (TIFF 429 kb) [file 11869_2022_1232_MOESM5_ESM.tiff]

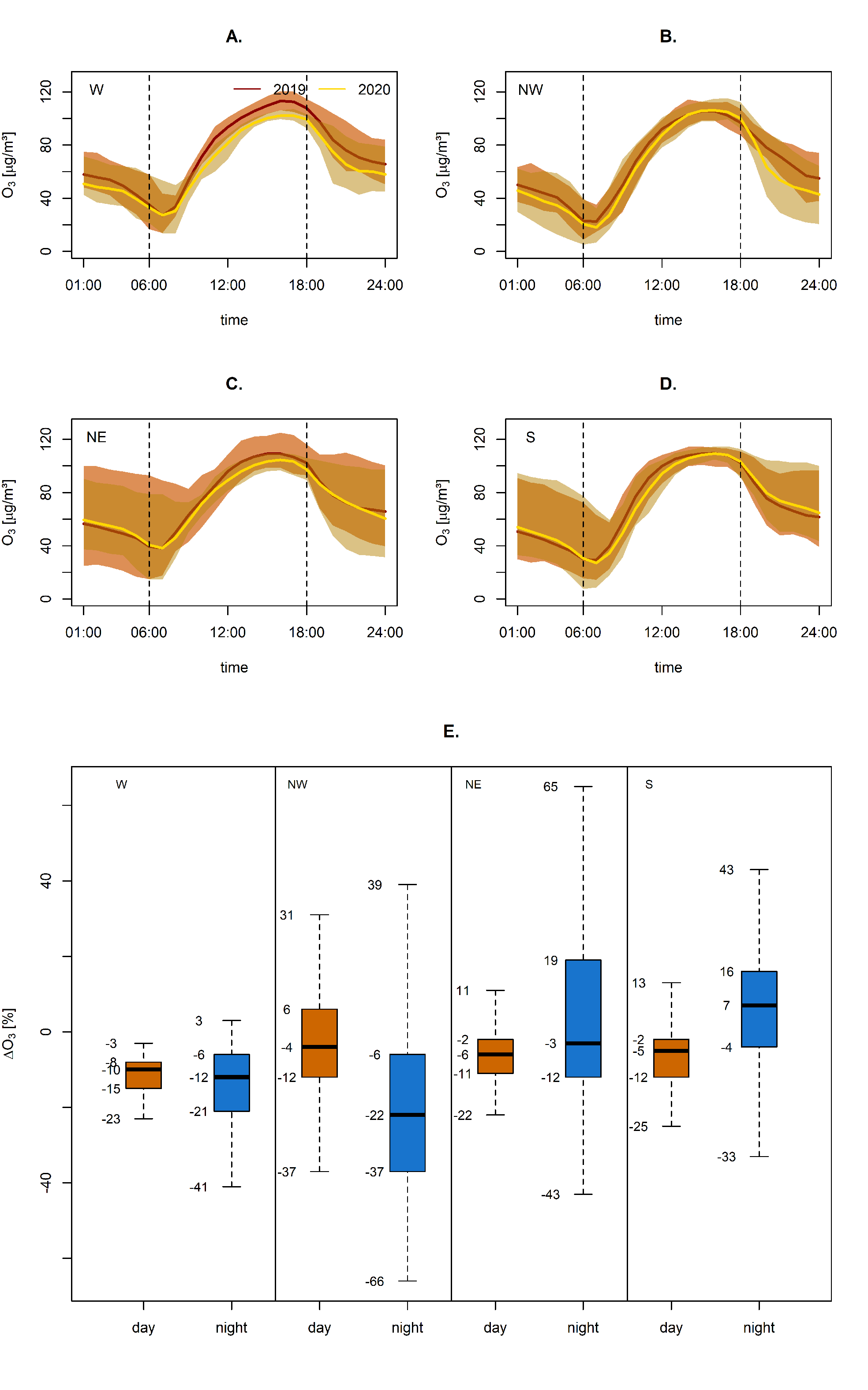

Supplement: Supplementary file 11 — Mean daily cycles of O3 at suburban/urban background monitoring sites for individual sectors (W (A), NW (B), NE (C) & S (D)). Red (2019) and yellow (2020) shadings indicate the range between maximum and minimum values. Bold lines indicate subdomain averages. Changes in day- (orange) and night-time (blue) variations of mean diurnal cycles relative to 2019 (E). Note, outliers (data points exceeding 1.5 times the interquartile range) have been omitted. All data is given as hourly averages. (PNG 69 kb) [file 11869_2022_1232_Fig14_ESM.png]

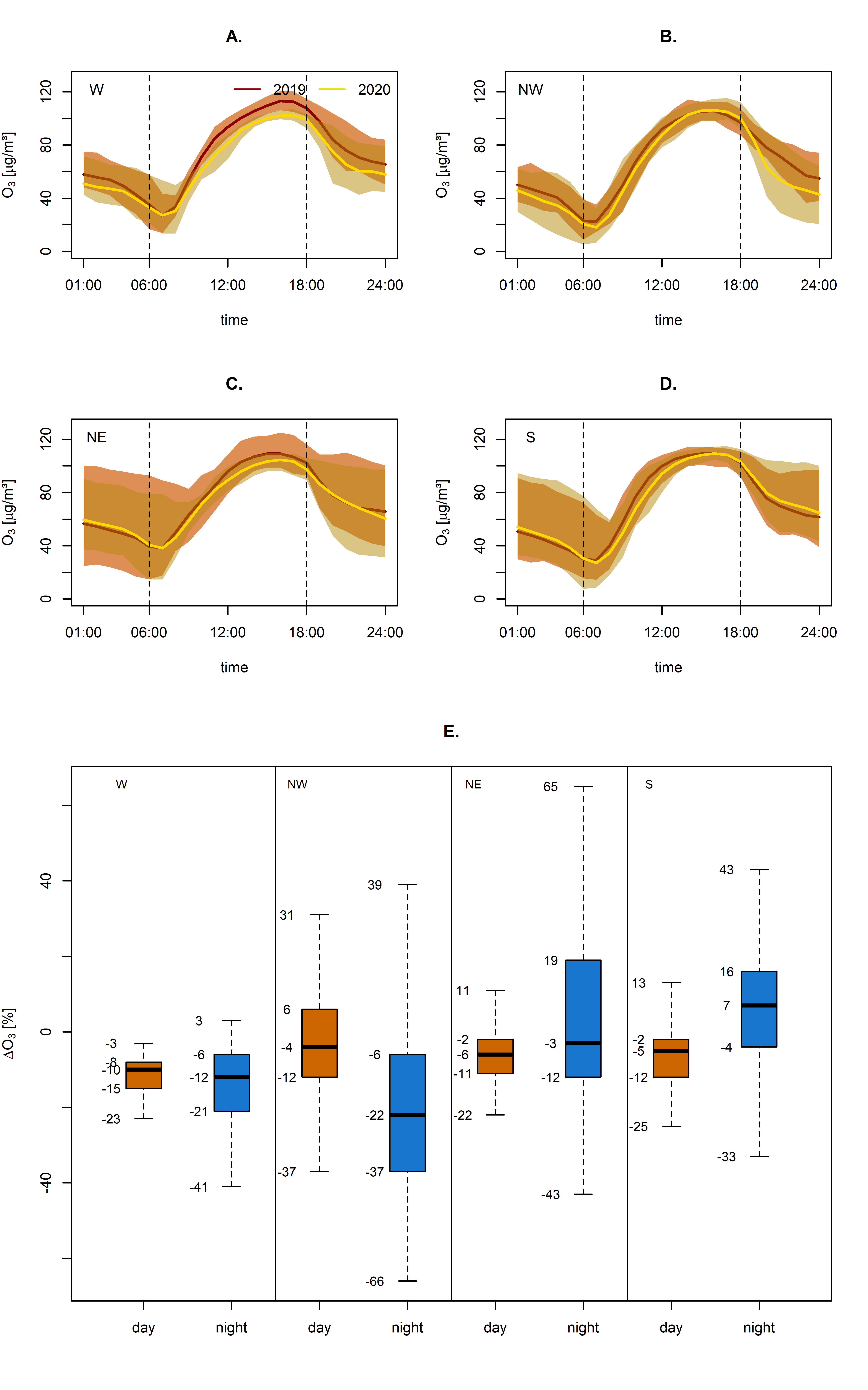

Supplement: Supplementary file 12 — High Resolution Image (TIFF 300 kb) [file 11869_2022_1232_MOESM6_ESM.tiff]

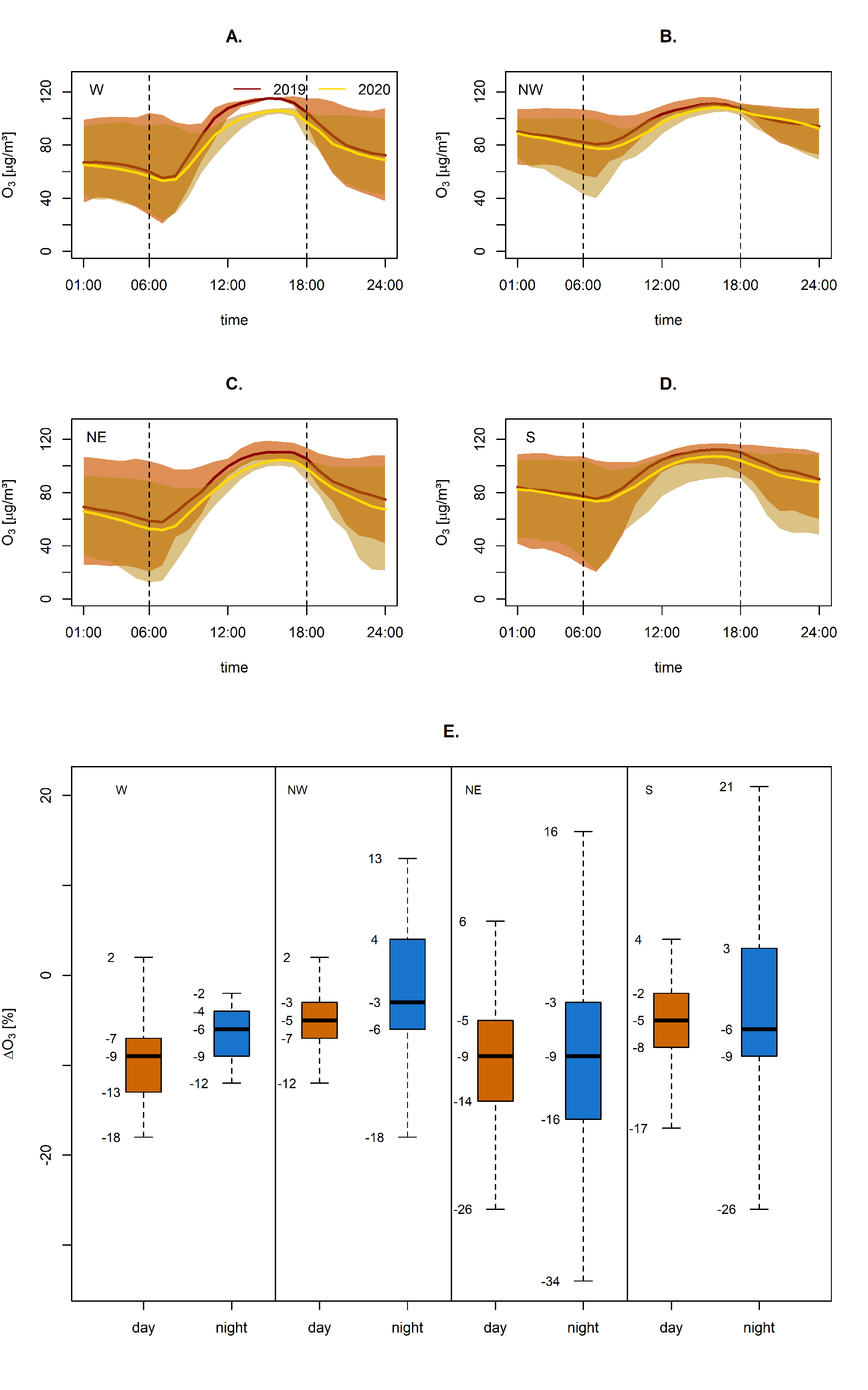

Supplement: Supplementary file 13 — Mean daily cycles of O3 at rural background monitoring sites for individual sectors (W (A), NW (B), NE (C) & S (D)). Red (2019) and yellow (2020) shadings indicate the range between maximum and minimum values. Bold lines indicate subdomain averages. Changes in day- (orange) and night-time (blue) variations of mean diurnal cycles relative to 2019 (E.). Note, outliers (data points exceeding 1.5 times the interquartile range) have been omitted. All data is given as hourly averages. (PNG 64 kb) [file 11869_2022_1232_Fig15_ESM.png]

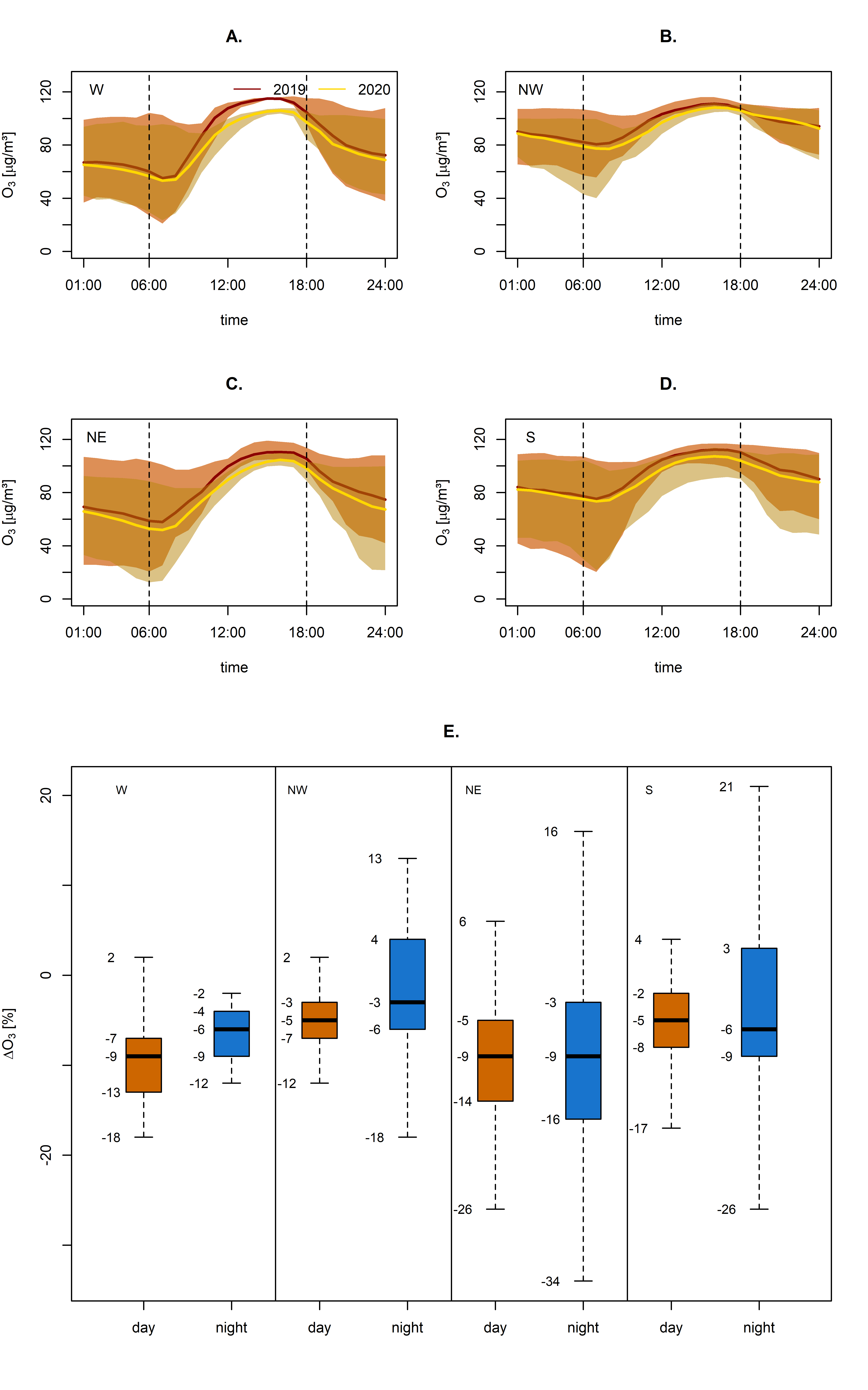

Supplement: Supplementary file 14 — High Resolution Image (TIFF 280 kb) [file 11869_2022_1232_MOESM7_ESM.tiff]
