# Supplementary material for: Quantifying changes in ambient NOx, O3 and PM10 concentrations in Austria during the COVID-19 related lockdown in spring 2020
Source: Air Qual Atmos Health. 2022 Jul 22;15(11):1993–2007. doi: 10.1007/s11869-022-01232-w (PMC9305063; doi:10.1007/s11869-022-01232-w)
Supplement: Supplementary file 15 — (DOCX 37 kb) [file 11869_2022_1232_MOESM8_ESM.docx]

Table S1: List of the employed sites of the Austrian air pollution monitoring network. Stations marked with a */† serve as background and traffic-influenced sites, respectively, for the PM analysis.

| **No** | **Station name** | **Code** | **Type** | **Area** | **Sector** | **Pollutants** | | |
| --- | --- | --- | --- | --- | --- | --- | --- | --- |
| 1 | Gärberbach A13 ^†^ | AT72223 | Traffic | Rural | W | NO_x_ |  | PM_10_ |
| 2 | Hall in Tirol ^†^ | AT72227 | Traffic | Suburban | W | NO_x_ |  | PM_10_ |
| 3 | Heiterwang * | AT72710 | Background | Rural | W | NO_x_ | O_3_ | PM_10_ |
| 4 | Imst A12 ^†^ | AT72315 | Traffic | Rural | W | NO_x_ |  | PM_10_ |
| 5 | Innsbruck - Reichenau | AT72106 | Traffic | urban | W | NO_x_ | O_3_ | PM_10_ |
| 6 | Innsbruck - Sadrach | AT72113 | Background | suburban | W | NO_x_ | O_3_ |  |
| 7 | Innsbruck - Zentrum ^†^ | AT72110 | Traffic | urban | W | NO_x_ |  | PM_10_ |
| 8 | Kramsach | AT72538 | Background | rural | W | NO_x_ | O_3_ |  |
| 9 | Kufstein ^†^ | AT72552 | Traffic | urban | W | NO_x_ |  | PM_10_ |
| 10 | Kundl A12 | AT72550 | Traffic | rural | W | NO_x_ |  |  |
| 11 | Vomp an der Leiten ^†^ | AT72822 | Traffic | rural | W | NO_x_ |  | PM_10_ |
| 12 | Vomp A12 Raststätte ^†^ | AT72821 | Traffic | suburban | W | NO_x_ |  | PM_10_ |
| 13 | Wörgl ^†^ | AT72530 | Traffic | suburban | W | NO_x_ | O_3_ | PM_10_ |
| 14 | Höfen | AT72705 | Background | rural | W |  | O_3_ |  |
| 15 | Kufstein | AT72547 | Background | suburban | W |  | O_3_ |  |
| 16 | Dornbirn | AT80807 | Background | urban | W | NO_x_ |  |  |
| 17 | Feldkirch | AT81919 | Traffic | urban | W | NO_x_ |  |  |
| 18 | Höchst | AT80709 | Traffic | suburban | W | NO_x_ |  |  |
| 19 | Lustenau Wiesenrain | AT80706 | Background | urban | W | NO_x_ | O_3_ |  |
| 20 | Lustenau Zollamt | AT80710 | Traffic | suburban | W | NO_x_ |  |  |
| 21 | Sulzberg - Gmeind | AT80503 | Background | rural | W | NO_x_ | O_3_ |  |
| 22 | Wald am Arlberg S16 | AT82801 | Traffic | rural | W | NO_x_ | O_3_ |  |
| 23 | Bludenz | AT82708 | Background | suburban | W |  | O_3_ |  |
| 24 | Amstetten | AT30101 | Background | suburban | NW | NO_x_ | O_3_ | PM_10_ |
| 25 | Heidenreichstein | AT30502 | Background | rural | NW | NO_x_ | O_3_ | PM_10_ |
| 26 | Kematen an der Ybbs | AT32604 | Background | rural | NW | NO_x_ | O_3_ | PM_10_ |
| 27 | St. Valentin, A1 | AT30104 | Traffic | rural | NW | NO_x_ | O_3_ |  |
| 28 | Annaberg | AT31102 | Background | rural | NW |  | O_3_ |  |
| 29 | Kollmitzberg | AT30103 | Background | rural | NW |  | O_3_ |  |
| 30 | Bad Ischl | AT4S125 | Background | suburban | NW | NO_x_ | O_3_ | PM_10_ |
| 31 | Braunau | AT4S156 | Background | suburban | NW | NO_x_ | O_3_ | PM_10_ |
| 32 | Enns Kristein - A1 | AT4S217 | Traffic | rural | NW | NO_x_ |  | PM_10_ |
| 33 | Grünbach bei Freistadt | AT4S108 | Background | rural | NW | NO_x_ | O_3_ | PM_10_ |
| 34 | Steyr | AT4S409 | Background | suburban | NW | NO_x_ | O_3_ | PM_10_ |
| 35 | Vöcklabruck | AT4S407 | Background | suburban | NW | NO_x_ |  | PM_10_ |
| 36 | Wels | AT4S406 | Background | urban | NW | NO_x_ | O_3_ | PM_10_ |
| 37 | Linz - A7 | AT4S415 | Traffic | urban | NW | NO_x_ |  | PM_10_ |
| 38 | Linz - Neue Welt ^†^ | AT4S416 | Background | urban | NW | NO_x_ | O_3_ | PM_10_ |
| 39 | Linz - Römerberg B139 ^†^ | AT4S431 | Traffic | urban | NW | NO_x_ |  | PM_10_ |
| 40 | Linz - Stadtpark ^†^ | AT4S184 | Background | urban | NW | NO_x_ | O_3_ | PM_10_ |
| 41 | Traun | AT4S404 | Background | suburban | NW | NO_x_ | O_3_ | PM_10_ |
| 42 | Hallein A10 ^†^ | AT52300 | Traffic | suburban | NW | NO_x_ |  | PM_10_ |
| 43 | Hallein B159 ^†^ | AT52000 | Traffic | urban | NW | NO_x_ |  | PM_10_ |
| 44 | Haunsberg | AT53055 | Background | rural | NW | NO_x_ | O_3_ |  |
| 45 | Salzburg - Lehener Park ^†^ | AT51200 | Background | urban | NW | NO_x_ | O_3_ | PM_10_ |
| 46 | Salzburg - Mirabellplatz ^†^ | AT51066 | Traffic | urban | NW | NO_x_ | O_3_ | PM_10_ |
| 47 | Salzburg - Rudolfsplatz ^†^ | AT51000 | Traffic | urban | NW | NO_x_ |  | PM_10_ |
| 48 | St. Johann im Pongau | AT54057 | Background | suburban | NW | NO_x_ | O_3_ |  |
| 49 | Zell am See | AT56072 | Background | suburban | NW | NO_x_ | O_3_ | PM_10_ |
| 50 | St. Koloman Kleinhorn | AT52055 | Background | rural | NW |  | O_3_ |  |
| 51 | Liezen | AT60177 | Background | suburban | NW | NO_x_ | O_3_ | PM_10_ |
| 52 | Grundlsee Tressensattel | AT60157 | Background | rural | NW |  | O_3_ |  |
| 53 | Enzenkirchen * | AT0ENK1 | Background | rural | NW | NO_x_ | O_3_ | PM_10_ |
| 54 | Zöbelboden | AT0ZOE2 | Background | rural | NW | NO_x_ | O_3_ | PM_10_ |
| 55 | Eisenstadt | AT10001 | Background | suburban | NE | NO_x_ | O_3_ | PM_10_ |
| 56 | Kittsee | AT10003 | Background | suburban | NE | NO_x_ | O_3_ | PM_10_ |
| 57 | Bad Vöslau | AT30201 | Background | suburban | NE | NO_x_ | O_3_ | PM_10_ |
| 58 | Biedermannsdorf | AT31406 | Traffic | suburban | NE | NO_x_ |  | PM_10_ |
| 59 | Dunkelsteinerwald | AT31701 | Background | rural | NE | NO_x_ | O_3_ |  |
| 60 | Forsthof am Schöpfl | AT30202 | Background | rural | NE | NO_x_ | O_3_ |  |
| 61 | Gänserndorf | AT30401 | Background | suburban | NE | NO_x_ | O_3_ | PM_10_ |
| 62 | Groß-Enzersdorf | AT30407 | Background | rural | NE | NO_x_ |  |  |
| 63 | Hainburg | AT30301 | Background | suburban | NE | NO_x_ | O_3_ | PM_10_ |
| 64 | Klosterneuburg B14 | AT30599 | Traffic | urban | NE | NO_x_ |  | PM_10_ |
| 65 | Klosterneuburg | AT30601 | Background | suburban | NE | NO_x_ | O_3_ |  |
| 66 | Krems | AT32501 | Background | urban | NE | NO_x_ | O_3_ | PM_10_ |
| 67 | Mannswörth - A4 | AT32702 | Traffic | suburban | NE | NO_x_ |  | PM_10_ |
| 68 | Mödling | AT31401 | Background | suburban | NE | NO_x_ | O_3_ | PM_10_ |
| 69 | Payerbach - Kreuzberg | AT31502 | Background | rural | NE | NO_x_ | O_3_ |  |
| 70 | Pöchlarn | AT31204 | Background | rural | NE | NO_x_ | O_3_ |  |
| 71 | Purkersdorf | AT30065 | Background | suburban | NE | NO_x_ | O_3_ |  |
| 72 | Schwechat | AT32701 | Background | suburban | NE | NO_x_ | O_3_ | PM_10_ |
| 73 | St. Pölten - Europaplatz | AT32302 | Traffic | urban | NE | NO_x_ |  | PM_10_ |
| 74 | St. Pölten – Eybnerstr. | AT32301 | Background | suburban | NE | NO_x_ | O_3_ | PM_10_ |
| 75 | Stixneusiedl | AT30302 | Background | rural | NE | NO_x_ | O_3_ | PM_10_ |
| 76 | Stockerau | AT30902 | Traffic | suburban | NE | NO_x_ |  | PM_10_ |
| 77 | Streithofen | AT31904 | Background | rural | NE | NO_x_ | O_3_ | PM_10_ |
| 78 | Traismauer | AT31703 | Background | rural | NE | NO_x_ |  | PM_10_ |
| 79 | Tulln | AT31901 | Background | suburban | NE | NO_x_ | O_3_ | PM_10_ |
| 80 | Vösendorf | AT31402 | Traffic | suburban | NE | NO_x_ |  |  |
| 81 | Wiener Neudorf | AT31413 | Traffic | suburban | NE | NO_x_ |  | PM_10_ |
| 82 | Wiener Neustadt | AT32401 | Background | suburban | NE | NO_x_ | O_3_ | PM_10_ |
| 83 | Wolkersdorf | AT30403 | Background | rural | NE | NO_x_ | O_3_ |  |
| 84 | Zwentendorf | AT31902 | Background | rural | NE | NO_x_ |  |  |
| 85 | Himberg | AT30603 | Background | rural | NE |  | O_3_ | PM_10_ |
| 86 | Irnfritz | AT30801 | Background | rural | NE |  | O_3_ |  |
| 87 | Mistelbach | AT31301 | Background | suburban | NE |  | O_3_ | PM_10_ |
| 88 | Ziersdorf | AT30701 | Background | rural | NE |  | O_3_ | PM_10_ |
| 89 | Vienna - A23 ^†^ | AT90A23 | Traffic | suburban | NE | NO_x_ |  | PM_10_ |
| 90 | Vienna - AKH ^†^ | AT90AKC | Traffic | urban | NE | NO_x_ |  | PM_10_ |
| 91 | Vienna - Belgradplatz ^†^ | AT9BELG | Traffic | urban | NE | NO_x_ |  | PM_10_ |
| 92 | Vienna - Floridsdorf | AT90FLO | Traffic | urban | NE | NO_x_ |  | PM_10_ |
| 93 | Vienna - Gaudenzdorf ^†^ | AT9GAUD | Traffic | urban | NE | NO_x_ |  | PM_10_ |
| 94 | Vienna - Hermannskogel | AT9JAEG | Background | suburban | NE | NO_x_ | O_3_ |  |
| 95 | Vienna - Hietzinger Kai | AT90MBA | Traffic | urban | NE | NO_x_ |  |  |
| 96 | Vienna - Hohe Warte | AT900ZA | Background | urban | NE | NO_x_ | O_3_ |  |
| 97 | Vienna - Kendlerstraße ^†^ | AT9KEND | Traffic | urban | NE | NO_x_ |  | PM_10_ |
| 98 | Vienna - Liesing | AT9LIES | Traffic | suburban | NE | NO_x_ |  | PM_10_ |
| 99 | Vienna - Lobau ^†^ | AT90LOB | Background | suburban | NE | NO_x_ | O_3_ | PM_10_ |
| 100 | Vienna - Schafberg | AT9SCHA | Background | suburban | NE | NO_x_ |  | PM_10_ |
| 101 | Vienna - Stadlau | AT9STAD | Background | urban | NE | NO_x_ |  | PM_10_ |
| 102 | Vienna - Stephansplatz | AT9STEF | Background | urban | NE | NO_x_ | O_3_ |  |
| 103 | Vienna - Taborstraße ^†^ | AT90TAB | Traffic | urban | NE | NO_x_ |  | PM_10_ |
| 104 | Vienna - Laaer Berg ^†^ | AT90LAA | Background | urban | NE |  | O_3_ | PM_10_ |
| 105 | Illmitz | AT0ILL1 | Background | rural | NE | NO_x_ | O_3_ | PM_10_ |
| 106 | Pillersdorf bei Retz * | AT0PIL1 | Background | rural | NE | NO_x_ | O_3_ | PM_10_ |
| 107 | Oberschützen | AT10002 | Background | rural | S | NO_x_ | O_3_ | PM_10_ |
| 108 | Klagenfurt 1, A2 | AT2KA61 | Traffic | suburban | S | NO_x_ |  |  |
| 109 | Klagenfurt 2, A2 | AT2F103 | Traffic | suburban | S | NO_x_ |  |  |
| 110 | Klagenfurt - Sterneckstr. | AT2KA71 | Background | urban | S | NOx | O_3_ | PM_10_ |
| 111 | Klagenfurt -Völkerm.Str. | AT2KA21 | Traffic | urban | S | NOx |  | PM_10_ |
| 112 | Obervellach | AT2SP10 | Background | rural | S | NO_x_ | O_3_ | PM_10_ |
| 113 | Spittal a. d. Drau | AT2SP18 | Background | suburban | S | NO_x_ | O_3_ | PM_10_ |
| 114 | St. Georgen | AT2WO35 | Background | rural | S | NO_x_ | O_3_ | PM_10_ |
| 115 | Villach | AT2VI12 | Traffic | urban | S | NO_x_ |  | PM_10_ |
| 116 | Wolfsberg | AT2WO15 | Traffic | urban | S | NO_x_ | O_3_ | PM_10_ |
| 117 | Klagenfurt - Kreuzbergl | AT2KA41 | Background | suburban | S |  | O_3_ |  |
| 118 | Ebenthal Zell | AT2M226 | Background | rural | S |  |  | PM_10_ |
| 119 | Wiesmath | AT32101 | Background | rural | S |  | O_3_ |  |
| 120 | Tamsweg | AT55032 | Background | suburban | S | NO_x_ | O_3_ | PM_10_ |
| 121 | Zederhaus | AT55018 | Traffic | rural | S | NO_x_ | O_3_ | PM_10_ |
| 122 | Bockberg ^†^ | AT60151 | Background | rural | S | NO_x_ | O_3_ | PM_10_ |
| 123 | Bruck an der Mur | AT60180 | Background | suburban | S | NO_x_ |  | PM_10_ |
| 124 | Deutschlandsberg | AT60195 | Background | suburban | S | NO_x_ | O_3_ | PM_10_ |
| 125 | Fürstenfeld | AT60198 | Background | suburban | S | NO_x_ | O_3_ | PM_10_ |
| 126 | Hartberg | AT60179 | Background | suburban | S | NO_x_ | O_3_ | PM_10_ |
| 127 | Hochgössnitz | AT60137 | Background | rural | S | NO_x_ | O_3_ |  |
| 128 | Judenburg | AT60118 | Background | suburban | S | NO_x_ | O_3_ | PM_10_ |
| 129 | Kapfenberg | AT60145 | Background | urban | S | NO_x_ |  | PM_10_ |
| 130 | Knittelfeld | AT60119 | Background | urban | S | NO_x_ |  | PM_10_ |
| 131 | Köflach | AT60106 | Background | urban | S | NO_x_ |  | PM_10_ |
| 132 | Leibnitz | AT60197 | Background | suburban | S | NO_x_ |  | PM_10_ |
| 133 | Leoben - Göss | AT60141 | Background | urban | S | NO_x_ |  | PM_10_ |
| 134 | Leoben - Zentrum | AT60143 | Background | urban | S | NO_x_ | O_3_ | PM_10_ |
| 135 | Masenberg | AT60156 | Background | rural | S | NO_x_ | O_3_ | PM_10_ |
| 136 | Mürzzuschlag | AT60194 | Background | suburban | S | NO_x_ | O_3_ | PM_10_ |
| 137 | Voitsberg | AT60107 | Background | suburban | S | NO_x_ | O_3_ | PM_10_ |
| 138 | Weiz | AT60178 | Background | suburban | S | NO_x_ | O_3_ | PM_10_ |
| 139 | Zeltweg | AT60114 | Background | suburban | S | NO_x_ |  | PM_10_ |
| 140 | Graz - Don Bosco ^†^ | AT60164 | Traffic | urban | S | NO_x_ |  | PM_10_ |
| 141 | Graz - Mitte ^†^ | AT60172 | Background | urban | S | NO_x_ |  | PM_10_ |
| 142 | Graz - Nord | AT60138 | Background | suburban | S | NO_x_ | O_3_ | PM_10_ |
| 143 | Graz - Ost ^†^ | AT60171 | Traffic | urban | S | NO_x_ |  | PM_10_ |
| 144 | Graz - Süd ^†^ | AT60170 | Background | urban | S | NO_x_ | O_3_ | PM_10_ |
| 145 | Graz - West ^†^ | AT60139 | Background | suburban | S | NO_x_ |  | PM_10_ |
| 146 | Arnfels - Remschnigg | AT60190 | Background | rural | S |  | O_3_ |  |
| 147 | Graz - Lustbühel ^†^ | AT60036 | Background | suburban | S |  | O_3_ | PM_10_ |
| 148 | Graz - Schloßberg | AT60018 | Background | urban | S |  | O_3_ |  |
| 149 | Lienz - Amlacherkreuzung | AT72910 | Traffic | urban | S | NO_x_ |  | PM_10_ |
| 150 | Lienz - Tristacher-See-Str. | AT72912 | Background | suburban | S | NO_x_ | O_3_ |  |
| 151 | Vorhegg | AT0VOR1 | Background | rural | S | NO_x_ | O_3_ | PM_10_ |
| 152 | Klöch* | AT0KLH1 | Background | rural | S | NO_x_ |  | PM_10_ |
| 152 | Klöch* | AT60185 | Background | rural | S |  | O_3_ |  |
