# Supplementary material for: Quantifying changes in ambient NOx, O3 and PM10 concentrations in Austria during the COVID-19 related lockdown in spring 2020
Source: Air Qual Atmos Health. 2022 Jul 22;15(11):1993–2007. doi: 10.1007/s11869-022-01232-w (PMC9305063; doi:10.1007/s11869-022-01232-w)
Supplement: Supplementary file 16 — (DOCX 14 kb) [file 11869_2022_1232_MOESM9_ESM.docx]

**Table S2** List of “A”-days selected for the analysis of NO_x_ and O_3_ concentrations per subdomain and year. Numbers on top of each column indicate the total amount of days fulfilling the MFM criteria per subdomain and year.

| **Sector W** | **Sector NW** | **Sector NE** | **Sector S** |
| --- | --- | --- | --- |
| 11 | 9 | 11 | 11 |
| 2017-03-03 | 2017-03-03 | 2017-03-03 | 2017-03-13 |
| 2017-03-16 | 2017-03-13 | 2017-03-16 | 2017-03-27 |
| 2017-03-27 | 2017-03-16 | 2017-03-17 | 2017-03-28 |
| 2017-03-30 | 2017-03-17 | 2017-03-27 | 2017-03-29 |
| 2017-03-31 | 2017-03-27 | 2017-03-28 | 2017-04-03 |
| 2017-04-12 | 2017-03-31 | 2017-04-10 | 2017-04-21 |
| 2017-04-24 | 2017-04-24 | 2017-04-25 | 2017-04-24 |
|  | | | |
| 8 | 10 | 10 | 7 |
| 2019-03-22 | 2019-03-21 | 2019-03-21 | 2019-03-21 |
| 2019-03-29 | 2019-03-22 | 2019-03-22 | 2019-03-22 |
| 2019-04-01 | 2019-04-02 | 2019-04-16 | 2019-04-16 |
| 2019-04-16 | 2019-04-16 | 2019-04-17 | 2019-04-17 |
| 2019-04-17 | 2019-04-17 | 2019-04-19 | 2019-04-19 |
| 2019-04-18 | 2019-04-18 | 2019-04-22 | 2019-04-22 |
| 2019-04-19 | 2019-04-22 | 2019-04-24 | 2019-04-25 |
|  | | | |
| 12 | 11 | 11 | 11 |
| 2020-03-16 | 2020-03-16 | 2020-03-16 | 2020-03-17 |
| 2020-03-18 | 2020-03-20 | 2020-03-19 | 2020-03-20 |
| 2020-03-19 | 2020-04-01 | 2020-03-20 | 2020-04-02 |
| 2020-04-01 | 2020-04-02 | 2020-04-01 | 2020-04-03 |
| 2020-04-02 | 2020-04-08 | 2020-04-02 | 2020-04-06 |
| 2020-04-06 | 2020-04-09 | 2020-04-07 | 2020-04-08 |
| 2020-04-07 | 2020-04-10 | 2020-04-08 | 2020-04-09 |
