# Supplementary material for: Quantifying changes in ambient NOx, O3 and PM10 concentrations in Austria during the COVID-19 related lockdown in spring 2020
Source: Air Qual Atmos Health. 2022 Jul 22;15(11):1993–2007. doi: 10.1007/s11869-022-01232-w (PMC9305063; doi:10.1007/s11869-022-01232-w)
Supplement: Supplementary file 17 — (DOCX 14 kb) [file 11869_2022_1232_MOESM10_ESM.docx]

**Table S3** List of “C”-days selected for the analysis of PM_10_ concentrations per subdomain and year. Numbers on top of each column indicate the total amount of days fulfilling the MFM criteria per subdomain and year.

| Sector W | Sector NW | Sector NE | Sector S |
| --- | --- | --- | --- |
| 8 | 9 | 7 | 8 |
| 2017-03-14 | 2017-03-14 | 2017-03-14 | 2017-03-14 |
| 2017-03-22 | 2017-03-22 | 2017-03-22 | 2017-03-22 |
| 2017-03-23 | 2017-03-23 | 2017-03-23 | 2017-03-23 |
| 2017-03-24 | 2017-03-24 | 2017-03-24 | 2017-03-24 |
| 2017-03-29 | 2017-03-29 | 2017-03-30 | 2017-03-30 |
| 2017-04-11 | 2017-03-30 | 2017-04-11 | 2017-04-11 |
|  | | | |
| 13 | 11 | 11 | 11 |
| 2019-03-19 | 2019-03-19 | 2019-03-12 | 2019-03-19 |
| 2019-03-25 | 2019-03-25 | 2019-03-19 | 2019-03-25 |
| 2019-03-26 | 2019-03-26 | 2019-03-26 | 2019-03-26 |
| 2019-04-04 | 2019-04-04 | 2019-03-28 | 2019-03-28 |
| 2019-04-11 | 2019-04-10 | 2019-04-04 | 2019-04-05 |
| 2019-04-12 | 2019-04-12 | 2019-04-05 | 2019-04-10 |
|  | | | |
| 6 | 6 | 6 | 7 |
| 2020-03-23 | 2020-03-23 | 2020-03-23 | 2020-03-18 |
| 2020-03-24 | 2020-03-24 | 2020-03-24 | 2020-03-23 |
| 2020-03-26 | 2020-03-26 | 2020-03-26 | 2020-03-24 |
| 2020-03-30 | 2020-03-30 | 2020-03-30 | 2020-03-26 |
| 2020-03-31 | 2020-03-31 | 2020-03-31 | 2020-03-30 |
| 2020-04-03 | 2020-04-03 | 2020-04-03 | 2020-03-31 |
