# Supplementary material for: Quantifying changes in ambient NOx, O3 and PM10 concentrations in Austria during the COVID-19 related lockdown in spring 2020
Source: Air Qual Atmos Health. 2022 Jul 22;15(11):1993–2007. doi: 10.1007/s11869-022-01232-w (PMC9305063; doi:10.1007/s11869-022-01232-w)
Supplement: Supplementary file 18 — (DOCX 14 kb) [file 11869_2022_1232_MOESM11_ESM.docx]

Table S4: Difference between maxima and minima of Q50 and Q90 for PM_10_ concentrations (µg/m³) for individual subdomains during pre-lockdown and lockdown periods in 2020 and 2019 (given in parenthesis). Bold numbers highlight smaller difference values.

|  | pre-lockdown | | | |
| --- | --- | --- | --- | --- |
| Quantile/Sector | W | NW | NE | S |
| Q50 | **6.6** (19.1) | **8.2** (18.9) | **7.3** (14.4) | **18.8** (20.4) |
| Q90 | **26.6** (37.6) | 34.0 (**30.2**) | **16.0** (16.5) | 31.1 (**11.4**) |
|  | lockdown | | | |
| Quantile/Sector | W | NW | NE | S |
| Q50 | **3.4** (6.8) | **7.6** (12.8) | 10.9 (**6.9**) | **5.3** (12.9) |
| Q90 | **8.3** (13.2) | **6.9** (20.8) | 11.6 (**10.2**) | **9.5** (28.3) |
